# Genome wide inherited modifications of the tomato epigenome by trans-activated bacterial CG methyltransferase

**DOI:** 10.1007/s00018-024-05255-7

**Published:** 2024-05-20

**Authors:** Bapatla Kesava Pavan Kumar, Sébastien Beaubiat, Chandra Bhan Yadav, Ravit Eshed, Tzahi Arazi, Amir Sherman, Nicolas Bouché

**Affiliations:** 1 Institute of Plant Sciences, Agricultural Research Organization, Volcani Center, Derech Hamacabim 68, Rishon Lezion, Israel; 2 INRAE, AgroParisTech, Institute Jean-Pierre Bourgin for Plant Sciences (IJPB), Université Paris-Saclay, 78000 Versailles, France; 3 Molecular Biology, Acrannolife Genomics Private Limited, Chennai, Tamilnadu 600035 India; 4 Department of Genetics, Genomics, and Breeding, NIAB-EMR, East Malling, East Malling, ME19 6BJ UK

**Keywords:** Epigenetics, Tomato, DNA hypermethylation, Bacterial methyltransferase, Gene body methylation

## Abstract

**Background:**

Epigenetic variation is mediated by epigenetic marks such as DNA methylation occurring in all cytosine contexts in plants. CG methylation plays a critical role in silencing transposable elements and regulating gene expression. The establishment of CG methylation occurs via the RNA-directed DNA methylation pathway and CG methylation maintenance relies on METHYLTRANSFERASE1, the homologue of the mammalian DNMT1.

**Purpose:**

Here, we examined the capacity to stably alter the tomato genome methylome by a bacterial CG-specific *M.SssI* methyltransferase expressed through the LhG4/pOP transactivation system.

**Results:**

Methylome analysis of *M.SssI* expressing plants revealed that their euchromatic genome regions are specifically hypermethylated in the CG context, and so are most of their genes. However, changes in gene expression were observed only with a set of genes exhibiting a greater susceptibility to CG hypermethylation near their transcription start site. Unlike gene rich genomic regions, our analysis revealed that heterochromatic regions are slightly hypomethylated at CGs only. Notably, some *M.SssI*-induced hypermethylation persisted even without the methylase or transgenes, indicating inheritable epigenetic modification.

**Conclusion:**

Collectively our findings suggest that heterologous expression of *M.SssI* can create new inherited epigenetic variations and changes in the methylation profiles on a genome wide scale. This open avenues for the conception of epigenetic recombinant inbred line populations with the potential to unveil agriculturally valuable tomato epialleles.

**Supplementary Information:**

The online version contains supplementary material available at 10.1007/s00018-024-05255-7.

## Introduction

Epigenetic variation is mediated by epigenetic marks such as cytosine DNA methylation which occurs in three different contexts CG, CHG, or CHH (H = A, T or C) in plants [25]. DNA methylation plays a critical role in silencing Transposable Elements (TEs) and regulating gene expression [33]. DNA methylation patterns are regulated by various physiological and developmental stimuli, including environmental stresses [2]. In plants, the establishment of DNA methylation, including at CG sites, occurs via the RNA-directed DNA Methylation (RdDM) pathway, which involves the DOMAINS REARRANGED METHYLTRANSFERASE2 (DRM2) enzyme. Methylation maintenance of CG sites mainly relies on METHYLTRANSFERASE1 (MET1), the plant homologue of the mammalian DNMT1 enzyme. CG methylation occurs in both TEs and genes, leading to the formation of gene body methylation. However, the exact function of gene body methylation is currently unknown. CHG and CHH sites are maintained by methylases like CHROMOMETHYLASE2 (CMT2), CMT3 and DRM2 [25]. Out of the three cytosine methylation contexts, the most frequent, heritable, and less influenced by environmental factors is the symmetric methylation of CGs. In tomato, 80% of the CG sites display methylation [7], whereas rice exhibits a 40% global methylation rate [20], and *Arabidopsis* 24% [6].

Epialleles are alternative epigenetic forms of a specific locus that can potentially influence gene expression and be inherited across generations [50]. Natural epialleles were identified in plants such as the tomato *COLORLESS NON-RIPENING* (*CNR*) impairing fruit ripening [35], *SP11* which is a *B. rapa* epiallele involved in self-incompatibility [41] or the *Lcyc* epiallele involved in flower symmetry of toadflax [8] in addition to several *Arabidopsis* epialleles [1, 9, 21]. Most studies of epigenetic variation in plants are based on stripping the methylation by chemicals such as 5-Azacytidine or genetic means (*i.e.* mutants) and using the hypomethylated plants as a source of variation to study gene function and isolate new epialleles [29]. Our comprehension of the mechanisms involved in the creation and maintenance of epialleles was greatly advanced by the creation of epigenetic Recombinant Inbred Lines (epiRILs) which were generated by crossing wild-type *Arabidopsis* accessions with DNA hypomethylated mutants like *met1* or *decreased DNA methylation1* (*ddm1*) [22, 39]. Still, most of the studies exploring the significance and function of epigenetic modifications have primarily employed *Arabidopsis* as a model plant and relayed on reduction of DNA methylation to generate novel epigenetic variations.

An alternative approach to generating novel plant epialleles is by introducing foreign methylases to induce methylation. This is grounded in the belief that the inherent biological processes will preserve these changes over time. Expression in tobacco of the *E. coli* dam methylase leads to high adenosine methylation at GATC sites and a set of biological phenotypes [47] demonstrating that a bacterial methylase can methylate plant DNA *in-vivo*. In another work, a foreign methylated DNA could be maintained into tobacco by the plant machinery [49], providing evidence that plants can recognize and maintain de novo methylated sites. *M.SssI* from the *Mollicutes spiroplasma* species is a bacterial methylase that catalyzes specifically CG methylation [40]. *M.SssI* was shown to be active in vitro, associated with Zinc Finger (ZF) proteins [4, 54], triple-helix-forming oligonucleotides [48] or catalytically-inactive Cas9 (dCas9) [28] and in vivo with dCas9 in *E. coli* [42, 53], mammalian cells [52], mouse oocytes or embryos [55] or with Transcription Activator-Like Effector (TALE) fusion proteins in mouse [56]. The ability of different *M.SssI* variants fused to a dCas9 to induce methylation in a specific locus was also demonstrated in *Arabidopsis* [13], as well as the potential of the newly acquired methylation to be inherited*.* The same *M.SssI* variant fused to an artificial ZF domain induces methylation in specific and nonspecific modes that were also inherited by the next generations [31]. However, till now, utilizing native *M.SssI* to induce genome scale CG methylation in plants was not reported. In this study, we overcame difficulties to express native *M.SssI* in tomato using a two-component transcription activation system [37]. Analysis of the methylome of the trans-activated plants expressing *M.SssI* revealed that the expression of *M.SssI* devoid of fusion proteins has significant repercussions on the overall methylation homeostasis of tomato even when the transgenes were segregated away in the following generations.

## Results

### Ectopic expression of a bacterial DNA methylase in tomato

*M.SssI* is a bacterial CG methyltransferase with specific codon usage [40]. To constitutively express *M.SssI in planta*, we optimized its codon usage and added a nuclear localization signal in-frame at the 3’-end. The potato *IV2* intron [10] was introduced into the plant-adapted *M.SssI* coding sequence to prevent bacteria from expressing the active enzyme (Figure S1) facilitating its cloning into a binary plasmid. The disarmed plant-adapted *M.SssI* (here after named *disM.SssI*) was cloned in front of a double *CaMV 35S* promoter followed by a TMV omega leader sequence to constitutively express it and assist its translation, respectively (Methods). To test whether the *disM.SssI* enzyme is active *in planta*, we transiently expressed it in *Nicotiana benthamiana* leaves and quantified the global cytosine methylation levels of their genomic DNA 2 days after infiltration (2 dpi). The *pART27_2* × *35S_Omega_disM.SssI* infiltrated leaves showed a significant increase in global 5-methyl cytosine compared to the empty vector infiltrated leaves (Figure S2). This result suggests that *disM.SssI* was active *in planta*. Transformation of *Arabidopsis* and tomato plants with *Agrobacterium* carrying the *pART27_2* × *35S_Omega_disM.SssI* binary plasmid, repeatedly failed to recover transgenic plants, suggesting that constitutive expression of *disM.SssI* might be lethal to plants.

**Fig. 1:**
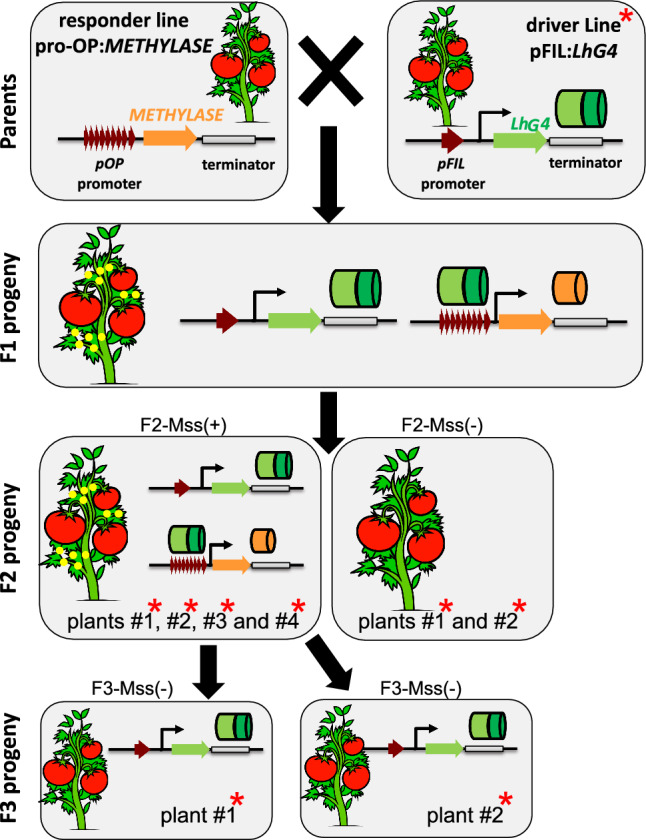
Schematic representation of the different plants produced in this study. Yellow sphere, expression of the *M.SssI* enzyme. Red asterisk, plant methylomes sequenced in this study

**Fig. 2:**
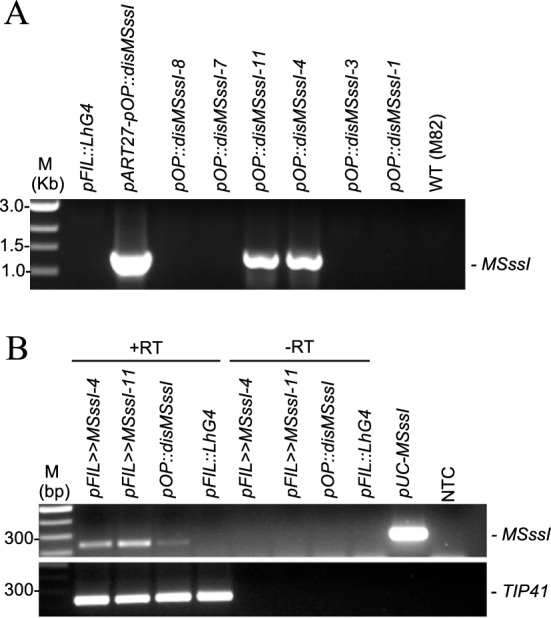
Transactivation of *pOP::disMSssI* results in its expression in tomato. **A** Genotyping of transgenic tomato plants (F1 generation) by PCR to test for the presence of the *pOP::disM.SssI* transgene. The plasmid *pART27-pOP::disM.SssI* was used as a positive control and the plasmid *pFIL::LhG4* construct as a negative control. Wild-type DNA (WT) correspond to DNA extracted from M82 cultivar leaves. M, DNA marker. **B** Expression of *disM.SssI* analysed by RT-PCR in two different F1 lines carrying both transgenes (*pFIL* >  > *pOPdisM.SssI-4 and 11)*. The absence of Reverse Transcriptase (-RT) was used for negative control and the *pUC::disM.SssI* plasmid DNA as a positive control. The *TIP41* gene was used as an equal loading control. M, DNA marker. NTC, No Template Control

To facilitate the expression of *M.SssI* in tomato, we utilized the LhG4/pOP transactivation system, which separates the transformation and transgene expression steps [37]. Two independent *pOP::disM.SssI* transgenic M82 responder lines, were obtained by transformation and regeneration (Methods). The *pOP* promoter is normally inactive and is *trans*-activated only in the presence of its artificial *pOP* activator *LhG4* (Fig. 1). To induce the expression of *disM.SssI*, the *pOP::disM.SssI* responder lines carrying the construct (Fig. 2A) were crossed with a homozygous *pFIL::LhG4* driver line expressing *LhG4* under the *FILAMENTOUS FLOWER* (*FIL*) promoter that was described as primordia and leaf specific [30]. The F1 transactivated progenies (*pFIL::LhG4* >  > *pOP::disM.SssI*) germinated normally and overexpressed the *disM.SssI* transgene (Fig. 2B). Although their cotyledons were not different from that of wild-type plants, some F1s developed severely distorted leaves consistent with the *pFIL* expression domain (Figure S3) and reminiscent of the tomato *wiry* phenotype [58]. All *pFIL* >  > *disM.SssI* plants were fertile and further analyses were done on their F2 progeny (Fig. 1).

### Expressing *M.SssI* increases the CG methylation of tomato genes

**Fig. 3:**
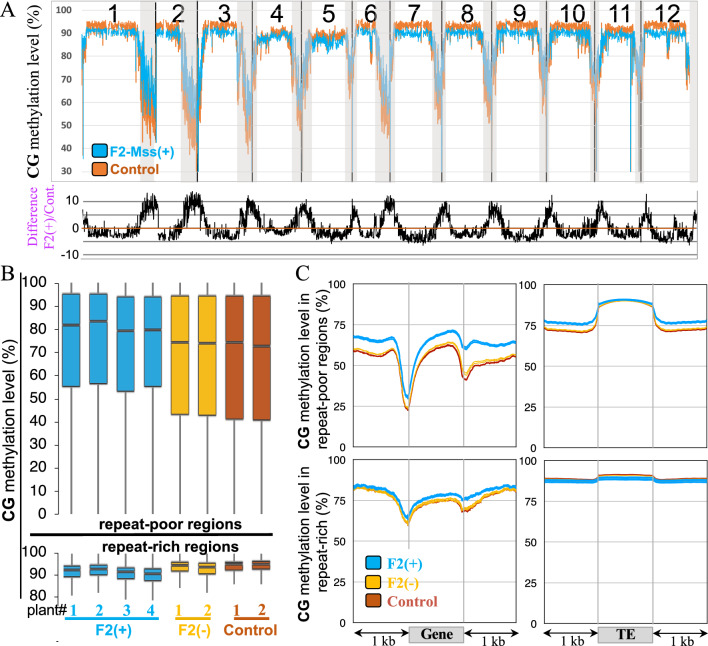
CG methylation in tomato F2 plants expressing or not *M.SssI*. **A** Methylation across the 12 chromosomes of tomato determined for non-overlapping 200 kb-bins that cover the entire genome. The methylation levels correspond to the proportions of methylated cytosines relative to the total number of cytosines calculated by aggregating the outcomes from all F2-Mss( +) or control plants. The regions enriched for genes are in grey. **B** Box plots showing mean methylation content of the F2-Mss( +), F2-Mss(-) and control lines. The SL2.50 version of the tomato genome assembly [46] was segmented in 1 kb windows; methylation levels correspond to the proportions of methylated cytosines relative to the total number of cytosines. Only cytosines covered by at least five reads were considered and only bins containing at least 10 valid cytosines were considered. The repeat-rich and repeat-poor regions were defined as previously described [23]. Control: *pFIL::LhG4* driver lines. The correlation network diagram constructed using Spearman’s correlation coefficients to illustrate the relationships among the CG methylation bins depicted in the boxplots is shown in Figure S6. **C** Metaprofiles of CG methylation for genes and Transposable Elements (TEs). TEs were annotated with REPET [12]

To study the impact of the expression of *M.SssI* on tomato genome methylation, the methylomes of plants expressing the transgene were sequenced. Genomic DNA was extracted from leaves of F2 progenies in which the transgenes were segregating to sequence the methylomes of four individual F2 plants carrying both the *pFIL:LhG4* and the *pOP::disM.SssI* transgenes (hereafter named F2-Mss( +) plants), as well as two other F2 sibling plants containing no transgenes (hereafter named F2-Mss(-) plants). The potential significance of methylome variation throughout transformation, across various generations, genotypes, and individuals cannot be understated, hence, it is crucial to reduce these variances through the utilization of a suitable control. We therefore conducted methylome sequencing for two individual *pFIL::LhG4* driver plants cultivated alongside the F2 plants, along with an additional two independent *pFIL::LhG4* driver plants grown at a separate instance. All the following analyses were carried out using these four control plants (hereafter named control driver line plants). The alignment of the reads with the sequences of the *pFIL::LhG4* or the *pOP::disM.SssI* transgenes reconfirmed that all plants belong to the different genotypes analysed (Figure S4). The levels of methylation per cytosine were determined for all methylation contexts (CG, CHG and CHH). On the chromosomal scale, DNA methylation was assessed by calculating methylation levels within 200 kb-windows that covered the entire genome. The results were then graphically represented by mapping them onto the 12 tomato chromosomes (Fig. 3A). Within the gene-containing regions of every chromosome, CG methylation was globally increased for the F2-Mss( +) plants expressing the bacterial methylase, compared to control driver line plants or F2-Mss(-) (Fig. 3A, grey areas and Figure S5A). In centromeric and pericentromeric regions enriched for TEs, the level of CG methylation exhibited a minor reduction for F2-Mss( +) plants but not for F2-Mss(-) plants (Fig. 3A, white areas and Figure S5). Methylation levels at chromosome scales were similar for the CHG contexts between F2-Mss(-), F2-Mss( +) and control driver line plants (Figure S5B and C). We also note that all F2 plants seem to be slightly hypermethylated in the CHH context compared to driver line plants (Figures S5D and E). These findings were confirmed when the average methylation levels were calculated within 1 kb-segments dividing the genome. Regions characterized by a high gene density and a low number of TEs (i.e. repeat-poor regions as described in [23]) were hypermethylated in the CG context for the four individual F2-Mss( +) plants, contrarily to the F2-Mss(-) or to the control driver line plants (Fig. 3B, *repeat-poor regions*; Figure S6). On the opposite, regions containing a low number of genes and densely populated by TEs (i.e. repeat-rich regions as described in [23]) were hypomethylated in the CG contexts only in F2-Mss( +) plants (Fig. 3B, *repeat-rich regions*; Figure S6). Therefore, methylome sequencings of leaves revealed a global increase of CG methylation in genic (repeat-poor) regions and a decrease of CG methylation in heterochromatic (repeat-rich) regions. Consequently, the introduction of the bacterial *M.SssI* enzyme into tomato resulted in a widespread disruption of the CG methylation balance.

On average, genes were hypermethylated in the CG context in F2-Mss( +) plants compared to F2-Mss(-) or driver line control plants (Fig. 3C). The differences in methylation were more pronounced in the 3’-end part of the genes and were observed for genes localized in both repeat-poor or -rich regions (Fig. 3C). No differences were observed in the CHG context (Figure S7). In the CHH context, genes localized in repeat-rich regions are more methylated in both F2-Mss( +) and F2-Mss(-) plants compared to controls (Figure S7). On the other hand, the methylation of TEs was similar between F2-Mss( +) and F2-Mss(-) plants, in all symmetric methylation contexts (Figure S8). Again, the CHH context is an exception and TEs have increased methylation compared to control driver lines, whether they are localized in heterochromatic or euchromatic regions (Figure S8). Altogether, the data show a specific increase of CG methylation in genes for plants expressing the bacterial *M.SssI* methylase.

### *M.SssI* targets unmethylated genic regions and accessible chromatin

**Fig. 4:**
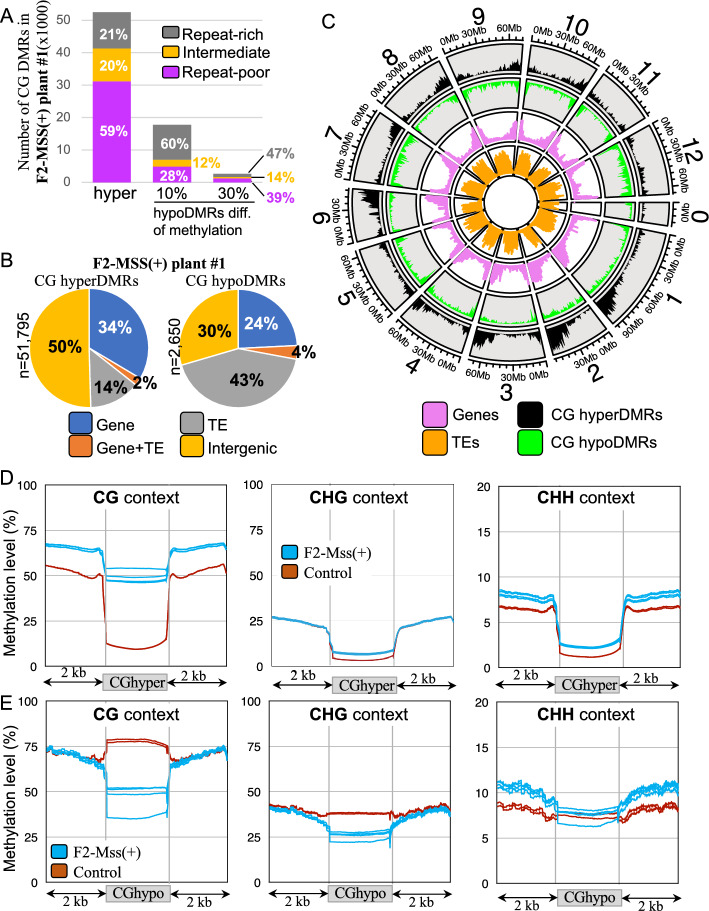
Nature and localization of the Differentially Methylated Regions (DMRs) identified between the F2-Mss( +) plant#1 are the driver line control plants for the CG methylation context. **A** Hypermethylated (*n* = 51,795) CG DMRs identified between the F2-Mss( +) plant#1 and the driver line control plants when a 30% absolute difference of methylation was applied and hypomethylated DMRs identified using a 30% (*n* = 2,650) or a 10% (*n* = 17,508) absolute difference of methylation. All other three F2-Mss( +) plants show similar numbers (Table S2 and Figure S9). The repeat-rich, repeat-intermediate and repeat-poor regions, based on the repeat densities, were defined as previously described [23]. **B** Nature of the CG hypomethylated and hypermethylated DMRs identified between the F2-Mss( +) plant#1 and the driver line control plants. “CDS + TE” are DMRs overlapping with both CDSs and transposons, “CDS”, DMRs overlapping with CDSs, and “TE” DMRs overlapping with Transposable Elements (TEs). All other DMRs were classified as “Intergenic”. The nature of the CG DMRs identified between all F2-Mss( +) plants and the driver line control plants is shown in Figure S10. **C** Densities of CG hypomethylated (*green*) and hypermethylated (*black*) DMRs identified between the F2-Mss( +) plant#1 and the driver line controls along the 12 tomato chromosomes. The density of genes and TEs are shown in pink and orange, respectively. **D** Metaprofiles of methylation in the three methylation contexts (CG, CHG, and CHH) for the CG hypermethylated and hypomethylated identified between the F2-Mss( +) plant#1 and the driver line control plants

The regions that were significantly differentially methylated (Differentially Methylated Regions, DMRs) between F2-Mss( +) or F2-Mss(-) plants and the driver line controls were identified. Compared to the driver line control plants, the F2-Mss( +) plants contained the highest number of DMRs with a vast majority of hypermethylated DMRs (hyperDMRs) in the CG context (Plant#1: n = 51,795; Plant#2: n = 48,467; Plant#3: n = 48,254; Plant#4: n = 54,758), consistent with *M.SssI* being active in these plants (see Fig. 4A for the example of F2-Mss( +) plant#1 and Figure S9 for all plants). CG hyperDMRs mainly overlapped with intergenic regions (50% of the CG hyperDMRs in F2-Mss( +) plant#1, Fig. 4B and Figure S10) and genes (34% of the CG hyperDMRs in F2-Mss( +) plant#1, Fig. 4B and Figure S10). In agreement with this last observation, 59% of the CG hyperDMRs of F2-Mss( +) plant#1 (n = 31,153) are found within repeat-poor regions enriched for genes, containing low amounts of repeats (Fig. 4A, *Repeat-poor regions*) and localized near chromosome arms (Fig. 4C). Moreover, ~ 34% of the tomato genes overlap with at least one CG hyperDMRs in the F2-Mss( +) plant#1 with similar results obtained for the three other plants analysed (32% for F2-Mss( +) plant#2, 33% for F2-Mss( +) plant#3 and 35% for F2-Mss( +) plant#4). The number and localization of the DMRs identified between the other F2-Mss( +) plants (#2, #3 and #4) and the controls are similar to what was found for F2-Mss( +) plant#1 (Table S2 and Figure S9). Altogether, our findings demonstrate that a significant portion of tomato genes undergo CG methylation when the corresponding plants ectopically express the bacterial *M.SssI* methylase.

Most of the CG hyperDMRs (96% n = 49,762) found between F2-Mss( +) plant#1 and the controls are not overlapping with hypermethylated or hypomethylated DMRs (hypoDMRs) in other cytosine contexts, namely CHG or CHH. Hence, most regions that experience an increase in CG methylation upon methylase expression do not undergo significant alterations in their methylation patterns for other types of DNA methylation. This was confirmed when the metaprofiles of methylation were drawn for the CG hyperDMRs of F2-Mss( +) plants (Fig. 4D, Figures S11 and s12). Indeed, CG hyperDMRs observed in F2-Mss( +) plants are indicative of regions where the control driver line plants exhibit minimal levels of basal methylation. On average, these regions are methylated at 10% for CGs, 3.7% for CHGs and 1.5% for CHHs in the control lines, reaching about 50% of CGs methylated in individual F2-Mss( +) plants with a significant (t-test p-value < 0.005) increase of 372% compared to the control (Fig. 4D, Figures S11 and S12). Both CHG and CHH sites gain methylation to a much lesser extent with a significant (t-test p-value < 0.005) increase of 90% for CHGs and 60% for CHHs (Fig. 4D, Figures S11 and S12). Altogether, the data indicate that the bacterial methylase targets preferentially euchromatic regions that were almost unmethylated in the wild-type tomato genome. In agreement with this hypothesis, we found that 44% (n = 23,014 for F2-Mss( +) plant#1) of the CG hyperDMRs overlap with accessible chromatin regions revealed genome-wide using ATAC-seq (Assay for Transposase-Accessible Chromatin using sequencing) data available for tomato meristem-enriched tissues [19]. Accessible chromatin regions correspond to 10% of the total genome sequence length [19].

There are almost 20 times less CG hypoDMRs (*n* = 2,650 for F2-Mss( +) plant#1) localized more predominantly in heterochromatin (Fig. 4A) and mostly matching TEs (Fig. 4B). In addition, CG hypoDMRs also seem to be slightly hypomethylated in the CHG but not in the CHH context (Fig. 4E). When the threshold of CG methylation difference was lowered from 30 to 10%, we observed a significant increase in the number of detected CG hypoDMRs (Plant#1: *n* = 17,508; Plant#2: *n* = 14,924; Plant#3: *n* = 26,189; Plant#4: *n* = 31,441). Between 56 and 68% of those hypoDMRs were found in repeat-rich regions (Fig. 4A), overlapping TEs. The results show a shift in CG methylation from heterochromatin to euchromatic regions, in agreement with chromosome scale observations (Fig. 3A).

### CG hyperDMRs are transmitted between tomato generations

**Fig. 5:**
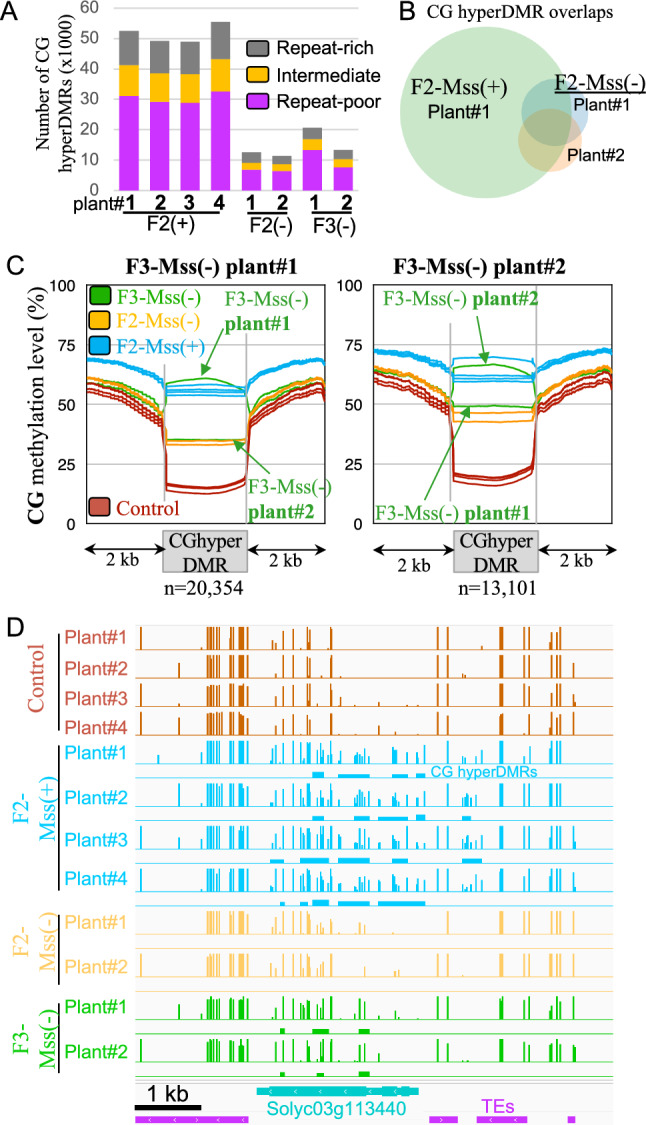
CG hypermethylated DMRs induced by *M.SssI* are transmitted between tomato generations. **A** Hypermethylated CG DMRs detected in F2 and F3 plants compared to the driver line control plants. The repeat-rich, repeat-intermediate, and repeat-poor regions, based on the repeat densities, were defined as previously described [23], using the SL2.50 version of the genome assembly. The numbers of DMRs for all plants are shown in Figure S9. **B** Overlap of hypermethylated CG DMRs between F2-Mss( +) plant#1, F2-Mss(-) plant#1 and F2-Mss(-) plant#2. **C** Methylation levels of plant#1 and plant#2 F3-Mss(-) CG hyperDMRs. The average methylation levels were determined by dividing the DMRs into 100-bp bins. Methylation levels in regions located 2 kb upstream and 2 kb downstream the DMRs are shown. **D** Example of genome view of CG methylation patterns in F2 and F3. CG hyperDMRs between the control driver line plants and the different genotypes are shown as colored rectangles below the methylation track

To ascertain whether the inheritance of CG hyperDMRs is possible across generations, we examined the methylomes of two F2-Mss(-) non transgenic plants, in which all transgenes from the F1 parent segregated away (Figure S4), and that were cultivated and subjected to sequencing alongside the F2-Mss( +) plants and inherited from the same F1. On average, the CG methylation of both genes and TEs was very similar genome-wide between the F2-Mss(-) non transgenic plants and the driver line controls (Figures S7 and S8). DMRs were identified, revealing that F2-Mss(-) plants still contain a high number of CG hyperDMRs (n = 12,410 for F2-Mss(-) plant#1 and n = 11,177 for F2-Mss(-) plant#2; Fig. 5A, Figure S9 and Table S2). By contrast, the number of CG hypoDMRs was much more limited (n = 745 for F2-Mss(-) plant#1 and n = 991 for F2-Mss(-) plant#2; Figure S9 and Table S2). As observed for the F2-Mss( +) plants, the CG hyperDMRs of F2-Mss(-) plants are mostly located within regions enriched for genes (n = 6,867 for F2-Mss(-) plant#1 and n = 4,452 for F2-Mss(-) plant#2; Fig. 5A). Furthermore, the CG hyperDMRs identified in F2-Mss(-) plants significantly coincide with the CG hyperDMRs present in their F2-Mss( +) counterparts. For instance, 77% of the CG hyperDMRs found in F2-Mss(-) plant#1 and 70% of the CG hyperDMRs found in F2-Mss(-) plant#2 overlap with CG hyperDMRs of F2-Mss( +) plant#1 (Fig. 5B). 44% of the CG hyperDMRs are shared among the two F2-Mss( +) plants analysed (Fig. 5B). This indicates that the vast majority of CG hyperDMRs detected in F2-Mss(-) non transgenic plants compared to the driver line controls are shared with their F2-Mss( +) transgenic sibling plants, implying that they were likely inherited from the F1 parent.

To track the potential transfer of CG DMRs across successive generations, F2-Mss( +) plant#3 and plant#4 were selfed and the F3 offspring was genotyped for the presence of the *pFIL:LhG4* and the *pOP:disM.SssI* transgenes. The F3 generation showed a classical Mendelian pattern of segregation for both transgenes, indicating that they existed in a heterozygous state in the preceding F2 parents. The methylomes of two F3 plants carrying solely the *pFIL:LhG4* transgene were sequenced and compared to the control driver line plants which are composed of a *pFIL:LhG4* set of plants including two plants grown together with these F3s and two other plants grown independently with F2s, as stated above. In both F3-Mss(-) plants, the CG hyperDMRs exhibited the highest count of DMRs (*n* = 20,354 for the F3-Mss(-) plant#1, a descendant of F2-Mss( +) plant#3 and *n* = 13,101 for the F3-Mss(-) plant#2, a descendant of F2-Mss( +) plant#4; Table S S2). Like for F2s, F3-Mss(-) CG hyperDMRs are localized mostly in genic regions (65% of the CG hyperDMRs for F3-Mss(-) plant#1 and 59% for F3-Mss(-) plant#2 are localized in regions containing low amounts of repeats; Fig. 5A, Figure S9 and Table S2). To better understand what the identified DMRs in the F3 correspond to, their methylation metaprofiles have been generated. The metaprofiles of CG hyperDMRs of F2-Mss( +) plants show that these regions are also methylated in both their F2-Mss(-) siblings and F3-Mss(-) progenies, but almost unmethylated in the control driver lines. No changes were detected in the two other CHG and CHH methylation contexts (Figures S11 and S12). CG hyperDMRs identified in F3-Mss(-) plants are at levels of methylation identical to the one of their F2-Mss( +) parents (Fig. 5C; Figure S13). Accordingly, most of the CG hyperDMRs of F3-Mss(-) plants are shared with their F2-Mss( +) parents (Fig. 5D). Indeed, 76% of the CG hyperDMRs of F3-Mss(-) plant#1 and 84% of F3-Mss(-) plant#2 overlap with CG hyperDMRs found in their corresponding F2-Mss( +) parents. These inherited DMRs account for 32% of the overall number of CG hyperDMRs found in F2-Mss( +) plant#3 and 20% of the CG hyperDMRs found in F2-Mss( +) plant#4.

Thus, both F2 and F3 plants lacking the *M.SssI* gene but descended from *pFIL* >  > *disMSssI* plants that express the transgenes, still carry CG hyperDMRs exhibiting retained levels of methylation when compared to their parent plants. This implies the inheritance of methylation patterns.

In agreement with the CHH methylation levels increase monitored in both F2-Mss( +) and F2-Mss(-) (Figures S5D, E, S6 and S7), many CHH hyperDMRs were identified between F2-Mss plants and control driver lines (Figure S9). Most of these DMRs overlap with TEs (for instance, 69.7% of the CHH hyperDMRs overlap with TEs in F2-Mss( +) plant #1) found within repeat-poor regions (Table S2). Nonetheless, F3-Mss(-) plants did not exhibit CHH hyperDMRs, suggesting a substantial divergence in the presence of CHH hyperDMRs between Mss(-) plants from distinct generations (F2 and F3). Moreover, CHH hyperDMRs are not overlapping with DMRs found for other methylation contexts (< 2% of the CHH hyperDMRs in F2-Mss( +) plant#1 overlap with other CG or CHG DMRs). Thus, hypermethylated CHH regions are largely independent of other methylation contexts and are specific of F2s. This implies that CHH hyperDMRs are likely independent of the *M.SssI* activity.

### Changes in CG methylation patterns correlate with limited effects on expression

**Fig. 6:**
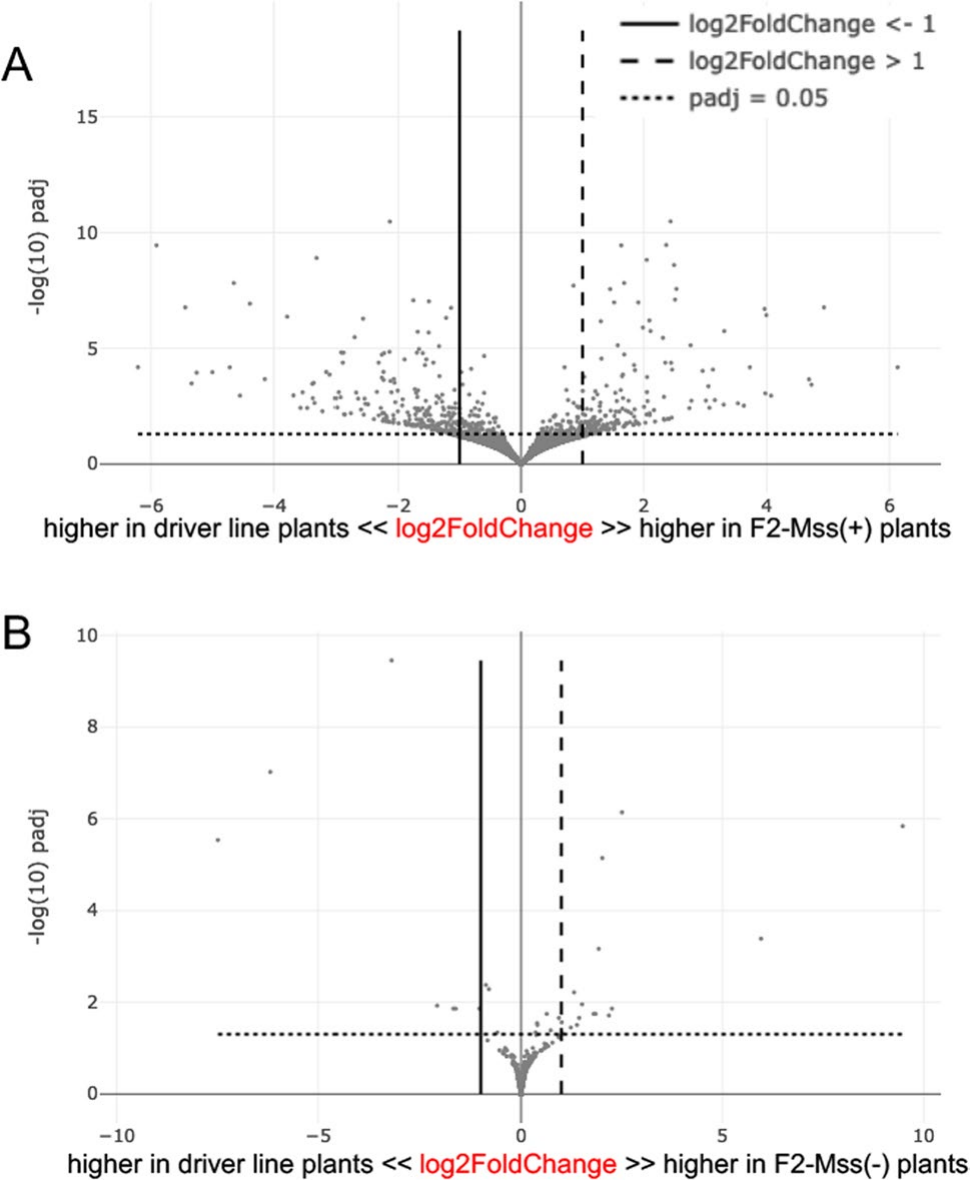
Differences of gene expression in F2-Mss( +) plants **A** and F2-Mss(-) plants **B** compared to the control lines, represented by volcano plots of -log(10) *p*-value against log(2) fold changes for Differentially Expressed Genes (DEGs)

To explore whether the accumulation of CG methylation within genes impacts their expression, we performed an RNAseq analysis of F2 plants. RNAs were extracted from leaves of three F2-Mss( +) plants, three F2-Mss(-) plants and three driver line plants grown together (Table S3). Reproducibility between biological replicates (a single replicate corresponds to an individual plant and is created by combining bulk samples of leaves) was confirmed by performing a Principal Component Analysis (PCA) to visualize the differences (Figure S14). Among the genes that were significantly differently regulated (adjusted *p*-value < 0.05 and log2FoldChange < -1 or > 1) between F2-Mss( +) and control driver line plants, 56% (*n* = 229) were downregulated (down Differentially Expressed Genes, downDEGs; Table S4; Fig. 6A) and 44% (*n* = 181) were upregulated (up Differentially Expressed Genes, upDEGs; Table S5; Fig. 6A). Altogether, up- and downDEGs in F2-Mss( +) plants represent only 1.2% of the tomato genes. This implies that CG hypermethylation changes occurring within or near genes in these plants have limited effects on global tomato gene expression. 48% to 51% of the downDEGs (n = 114 for F2-Mss( +) plant#1, *n* = 117 for plant#2, *n* = 109 for plant #3 and *n* = 117 for plant #4) overlap with CG hyperDMRs. By contrast, these numbers drop to 31 to 38% for the upDEGs (*n* = 69 for F2-Mss( +) plant#1, *n* = 65 for plant#2, *n* = 57 for plant#3 and *n* = 66 for plant#4) which is comparable to the average numbers of genes overlapping with at least one CG hyperDMR genome-wide in F2-Mss( +) plants (~ 35%). Only a maximum of 12% of the upDEG and 15% of the downDEGs overlap with CHG or CHH DMRs. By contrast, expression analyses of the three F2-Mss(-) plants revealed very few changes compared to control driver lines with only 16 upDEGs and 7 downDEGs (Fig. 6B). Thus, downregulated genes in F2-Mss( +) plants appear to exhibit a higher susceptibility to CG hypermethylation compared to upregulated genes.

**Fig. 7:**
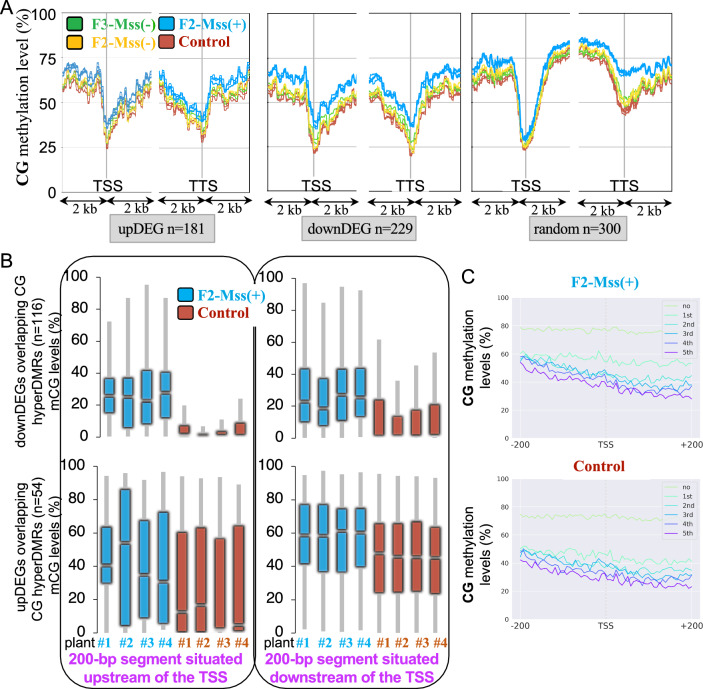
Methylation levels of Differentially Expressed Genes (DEGs) that are up- or downregulated (> ou < 1 log2FC) in F2/F3 plants expressing *M.SssI* (*Mss(* +*)*) or not (*Mss(-)*) versus the control driver line plants. The average methylation levels of the DEG was calculated by dividing the DEG region into 100-bp bins. Regions located 2-kb upstream and 2-kb downstream the DEGs are shown. Random: set of 300 genes selected randomly. **B** Box plots showing the mean methylation near the Transcription Start Site (TSS) of upregulated DEGs (upDEGs) overlapping with CG hyperDMRs. Only genes with TSS overlapping (± 500 bp) with CG hyperDMRs were considered. Only genes whose TSS overlap with CG hypermethylated regions within a range of ± 500-bp were considered. Methylation levels were calculated in regions upstream (-200 bp) and downstream (+ 200 bp) of F2-Mss( +) and control line TSSs as the proportions of methylated cytosines over the total number of cytosines. Only cytosines covered by at least five reads were considered and only bins containing at least 5 valid cytosines were kept. **C** Average methylation level profiling according to different expression groups around the TSS (± 200 bp) of F2-Mss( +) and control lines. Genes are grouped as non-expressed genes and five quantiles of expressed genes according to the gene expression level groups from low to high; the first quintile is the lowest, and the fifth is the highest

To further test this hypothesis, we analyzed the metaprofiles of DEGs for DNA methylation. The methylation profiles of downDEGs differ from those of upDEGs, or genes chosen randomly, mainly around the Transcription Start Site (TSS) and only for CG methylation (Fig. 7A and Figure S15). DEGs with CG hyperDMRs localised around their TSS (within a TSS distance of ± 500-bp) in at least one of the F2-Mss( +) plants were further analysed (n = 116 for downDEGs and n = 54 for upDEGs). Our findings revealed that the 200 bp region located upstream of the TSSs of downDEGs exhibited nearly negligible CG methylation in control lines, in contrast to F2-Mss( +) plants (Fig. 7B). The regions localised 200-bp downstream of the TSS of downDEGs were slightly more methylated (Fig. 7B). In contrast, the methylation patterns surrounding the TSSs of upDEGs were distinct, showing significantly elevated average levels of CG methylation preceding and following the TSS in both F2-Mss( +) and control plants (Fig. 7B). This suggests a correlation between the presence of CG methylation within the region adjacent to the TSS and the transcriptional decrease of the associated genes in F2-Mss( +) plants. Afterwards, the genes were categorized into two groups: non-expressed genes and expressed genes divided into five quantiles based on their gene expression levels, ranging from low to high. The lowest expression level corresponds to the first quintile, while the highest expression level corresponds to the fifth quintile. Metaprofiles were generated for the various gene categories within windows that cover a range of ± 200 bp around the TSS (Fig. 7C). A significant increase in methylation was only detected in the CG context, particularly in genes characterized by low expression levels (genes in the first quintile; Fig. 7C and Figure S16). Hence, the genes expressed at lower levels in the wild-type exhibit a higher sensitivity to CG hypermethylation around their TSS.

TEs found in the vicinity of genes can potentially interfere with gene expression [3]. However, the results reveal that the proportion of TEs overlapping with DEG (for upDEGs, *n* = 75 which represent 41.4% of all DEGs and for downDEGs *n* = 111 which represent 48.5% of all DEGs) follows a similar pattern to what is observed across the entire genome for all the genes (49.5%). This indicates that the genes differently transcribed in F2-Mss( +) plants are not particularly enriched for TEs compared to other genes in the tomato genome. The transcription of TEs was also examined, using the RNAseq data, to determine whether TEs are deregulated in F2-Mss plants (adjusted *p*-value < 0.05 and log_2_FoldChange < -1 or > 1). No TEs were found to be deregulated in F2-Mss( +) plants compared to the control driver lines and only one TE was downregulated in F2-Mss(-) plants. Employing identical bioinformatic procedures for comparison purposes, 7,783 TEs were found to be deregulated in *Slddm1* plants [7] with almost 92% that were upregulated, which is in agreement with the function of DDM1 in promoting the maintenance of DNA methylation. Thus, when the bacterial methylase is expressed, only a very small fraction of TEs become deregulated in comparison to the total number of tomato TEs.

## Discussion

Here, we expressed the bacterial CG-specific *M.SssI* methyltransferase under the control of the *CaMV 35S* promoter in tomato using the LhG4/pOP transactivation system, which separates the transformation and transgene expression steps. The plants expressing the methyltransferase are specifically hypermethylated in the CG context, in accessible chromatin regions, and thus mostly in genes. Conversely, heterochromatic regions are slightly hypomethylated in the CG context only. We also demonstrate that CG hyperDMRs produced by *M.SssI* can be inherited in the absence of bacterial methylase.

*M.SssI* fused to a ZF protein was introduced in *Arabidopsis* previously [13, 31]. Even after multiple attempts, we were unsuccessful in directly introducing a *M.SssI* gene cloned in front of a double *CaMV 35S* promoter into *Arabidopsis* or tomato through transformation. Instead, the LhG4/pOP transactivation system [37] was successfully used in tomato. The variation with previous outcomes achieved in *Arabidopsis* may be attributed to the strength of the promoter used and therefore differences in *M.SssI* expression levels. Indeed, we employed a constitutive strong *CaMV 35S* promoter, while previous experiments were conducted with a *M.SssI*-ZF fusion driven by a *UBQ10* promoter [31], which is recognized for its ability to enable moderate expression in virtually all tissues of *Arabidopsis* [15]. An alternative explanation for the differences observed might be due to the experimental approaches. The *M.SssI*-ZF fusion protein exploited in *Arabidopsis* which was initially designed to target and bind to two neighboring repeats within the *FWA* promoter, demonstrated a broader binding capacity, affixing and functioning on numerous off-target sites. However, it is unclear whether the *M.SssI*-ZF fusion could bind without restriction to off-target sites or if those sites possess distinct features that are specifically recognized by the *M.SssI*-ZF fusion protein. The *M.SssI* used in this study was free of any fusion protein, therefore potentially preserving its capacity to bind a wider array of accessible target regions, potentially including novel targets, in comparison to the *M.SssI*-ZF fusion. Those targets may exhibit a heightened susceptibility to hypermethylation. In this study, we demonstrate that the *M.SssI* prokaryotic methyltransferase is active in tomato, a model crop, opening the door to targeted CG methylation as has already been demonstrated for *Arabidopsis* [13] or mice embryos [56]. Moreover, our results suggest that a new type of epiRILs [22, 39] can be generated by overmethylating DNA instead of stripping methylation, which could result in new epialleles and traits in crops. Plants expressing the bacterial methylase *M.SssI* show an overall modification of CG methylated sites. Methylation levels are more specifically increased within chromosome arms enriched for genes, and a small but significant decrease of CG methylation was detected in pericentromeric regions that are densely populated with repeats (Fig. 3). In a recent study, we observed similar changes of DNA methylation homeostasis in the tomato *ddm1* mutants [7]. DDM1 is a chromatin remodeler essential for maintaining DNA methylation and histone epigenetic marks, particularly in heterochromatic regions [27, 34]. In the *ddm1* mutant of tomato, the RdDM is partially redirected from euchromatin towards heterochromatin [7]. Consequently, an imbalance in DNA methylation homeostasis occurred, marked by a reduction in both siRNAs and CHH methylation in chromosome arms and a parallel increase in heterochromatic regions. Thus, the RdDM pathway components appear to be diluted throughout the genome in *ddm1* tomato cells and certain elements of this pathway, such as enzymes or metabolites, may be limited in their availability. Other groups have obtained similar results with the *ddm1* mutants from rice [44]. In this study, we expand upon this observation to show that the steady-state levels of CG methylation are also adjusted genome wide in tomato. Two possible hypotheses could explain the disturbance in CG methylation balance in plants expressing the bacterial CG-specific methylase. Firstly, the main endogenous enzyme responsible for maintaining CG methylation in plants, MET1, could be at limiting production to preserve the overall CG methylated sites including the one newly introduced by *M.SssI* along with the highly abundant CG methylated sites that are consistently present in heterochromatic regions. Secondly, the cell might not produce enough metabolites required by the DNA methyltransferases. The methylation of DNA requires S-adenosylmethionine, a universal methyl-group donor, as a cofactor, which is generated through the methionine cycle. *Arabidopsis* mutants impaired in the methionine cycle, like *mthfd1-1* [16], *methionine adenosyltransferase4* [36] or *methionine synthase1* [57] show decreased DNA and histone methylation, along with TE activation. It is therefore possible that S-adenosylmethionine is a limiting factor in *M.SssI* expressing plants, leading to the changes observed between CG methylation of euchromatin and heterochromatin when the bacterial methylase is active (Fig. 3A and 3B).

Numerous studies have pointed out a modest correlation between changes in DNA methylation profiles and shifts of gene expression in plants [14]. The expression of the *M.SssI* bacterial methylase in tomato leads to a massive hypermethylation of genes in the CG context conducting to few changes in gene expression. Indeed, we found only 229 downregulated genes and 181 upregulated genes between plants expressing the methylase and the controls (Fig. 6), suggesting that the changes in gene expression are not widespread across the genome. Still, an association between CG hypermethylation and transcriptional repression of genes was observed when genes were hypermethylated in the CG context near their TSS (Fig. 7). Our findings revealed that genes exhibited a greater susceptibility to CG hypermethylation in the TSS region when they are expressed at lower levels in the wild type (Fig. 7C). A recent study demonstrates that specific genes are particularly susceptible to alterations in CG gene body DNA methylation [26]. Loss of *DDM1* in *Arabidopsis* not only reduces DNA methylation, but also enhances resistance to a biotrophic pathogen when combined with mild chemical priming. The overall decrease in gene body methylation in the *ddm1* mutant additionally hyperactivates some stress-responsive genes leading to plant resistance [26]. Like many other genes, stress response genes are hypomethylated in a *ddm1* background but they become transcriptionally active only when the plants are attacked by a pathogen [26]. Therefore, modulating CG DNA methylation at specific genes weakly expressed might be a way to fine tune their regulation. The function of gene-body methylation, if any, remains enigmatic and our study extends to crops the observations of Liu et al. [31] by showing that global elevation of CG gene body methylation (Fig. 3C) has minimal impact on the overall level of gene expression (Fig. 6).

While we did observe a substantial quantity of CG hyperDMRs in F2-Mss( +) plants, those are somatic epimutations as DNAs analyzed were extracted from leaves. Transmission of the newly acquired methylation patterns to the next generations relies on the activity of *M.SssI* in the Shoot Apical Meristem (SAM), and more specifically in stem cells that serve as a functional germline. However, in tomato, the Arabidopsis *pFIL* promoter that we used to drive the expression of the *M.SssI* bacterial methyltransferase seems to be specific of leaf primordia, and significant expression within the SAM was not detected by using GUS or GFP reporters [30]. Nevertheless, we found that 20 to 32% of the CG hyperDMRs were transmitted between F2 plants expressing the methylase and their F3 progenies in which the transgene carrying the *M.SssI* gene is absent. Thus, CG methylation is likely transmitted to the SAM by an indirect mechanism that needs to be deciphered. Alternatively, the *pFIL* promoter might be active at very low rates in SAMs, possibly explaining why the number of DMRs transmitted to the next generation is lowered compared to *Arabidopsis M.SssI*-expressing plants where 50 to 90% of the DMRs are inherited [31]. We also found that CG hyperDMRs newly appearing when the bacterial methylase is expressed are majorly not associated with CHG or CHH DMRs in both F2s and F3s. This is surprising considering that the RdDM pathway presumably triggers CHG and CHH methylation when methylation occurs [59]. Although they are few overlaps between CG hyperDMRs and DMRs found in other contexts, F2-Mss( +) CG hyperDMRs are slightly but significantly (t-test p-value < 0.001) hypermethylated in the CHG and CHH contexts (Fig. 4D; Figures S11 and S12). In the following F3-Mss(-) generation that inherited 20 to 30% of these CG hyperDMRs, the slight increase in both CHG and CHH methylation remains at similar levels, with no additional increase (Figure S13). Therefore, we do not observe between F2 and F3 generations an enrichment of CG hyperDMRs in other forms of methylation. The effectiveness of RdDM might be hindered by a relatively low number of generations in our experiment. Alternatively, a recent study [5] demonstrated that the absence of CG methylation and histone H1 (*h1met1* mutants) led to a rise in methylation at CHH instead of the anticipated decrease. This finding suggests that CG methylation is not the crucial chromatin marker for the RdDM-dependent methylation observed at heterochromatic TEs in *h1* mutants. Instead, the authors suggest that CHG/CHH methylation serves as the main marker for attracting the RdDM machinery, with H3K9 methylation-dependent mechanisms playing a secondary role [5].

In conclusion, expressing the bacterial *M.SssI* methylase devoid of any fusion proteins via trans-activation has drastic consequences on the overall CG methylation homeostasis in tomato. CG DNA hypermethylation that is triggered in one generation through the expression of a foreign methyltransferase can be passed down to the subsequent generation. This opens possibilities for engineering precise DNA methylation in tomato plants. Activation of the *pOP*:*:M.SssI* responder line generated in this study by other driver lines with distinct cellular, tissue and organ specificities will facilitate the targeted modification of their epigenomes. This could further broaden the range of inherited epigenetic variations generated in this study. The resultant epigenetic variation could allow for the creation of new epiRIL populations. These populations have the potential to reveal previously unknown phenotypes that might not be identified solely by studying epigenetic variations in natural accessions.

## Methods

### Plant material and growth conditions

The tomato (*S. Lycopersicum*) cv. M82 driver line *pFIL::LhG4* was described [30]. Germination and seedling growth took place in a growth chamber with a 16 h light period and 8 h dark period (photosynthetic photon flux density: 50 to 70 μmol m^−2^ s^−1^) at a constant temperature of 24 °C. For crosses, closed flowers were emasculated by removal of the petals and stamens and hand-pollinated with the pollen of an appropriate homozygous driver line. Seeds were surface sterilized by treatment with 70% ethanol for 2 min followed by 3% sodium hypochlorite for 15 min. After rinsing three times with sterile distilled water, seeds were sown on MS culture medium with or without antibiotics. Germination and seedling growth were done in a growth chamber with a 16 h light/8 h dark period at a constant temperature of 24 °C. Transgenic plants were moved and grown in 400 mL pots under greenhouse conditions with the temperature between 15 and25 °C in a peat mix with nutrients.

### Generation of tomato plants expressing *M.SssI*

Codon optimized bacterial methylase *M.SssI* with 2 × 35S promoter TMV omega, NLS and NOS terminator was synthesized (*Integrated DNA Technologies*, USA) and cloned into *pUC57* to create *pUC_M.SssI*. The potato *ST-LS1* IV intron [10] was amplified (*Infusion*, Takara Bio) using *M.SssIN_IV* forward and *M.SssIC_IV* reverse primers (Table S6) and cloned in the coding region of the methylase resulting in the *pUC_M.SssI_IV* plasmid carrying a disarmed plant adapted *M.SssI* (hereafter named *disM.SssI*). The methylase cassette was further subcloned in a binary vector *pART27* using the *Not*I restriction enzyme to yield *pART27_disM.SssI*. To clone under Op array, methylase was amplified using *M.SssI_HindIII* forward and reverse primers (Table S6) cloned into *pGEMT-Easy* (*Promega*, USA) and sequenced for verification. The methylase cassette was further sub-cloned in a binary vector *pART27OP::P19HA* [43] vector digested with *HindIII* replacing *P19HA* to result in *pART27_OP::disM.SssI*.

The cotyledons of 14 days old tomato cv. M82 were transformed by co-cultivation with *Agrobacterium* strain GV3101 carrying the binary vector *pART27-OP::disMSssI* as described previously [18]. Transgenic plants were selected on MS culture medium supplemented with Kanamycin (*Sigma*, USA). Presence of the transgene *pOP::disMSssI* was confirmed by PCR amplification on genomic DNA from plants that grew on selective media using *M.SssI-PCR* forward and *M.SssI-PCR* reverse primers (Table S6). For crosses, pollen from the *pFIL:LhG4* diver line was used to hand pollinate the emasculated flower. F1 progenies were genotyped for the presence of *pOP::disMSssI* and *pFIL::LhG4* transgenes by PCR using *M.SssI-PCR* forward, *M.SssI-PCR* reverse primers and LhG4-forward, LhG4-reverse primers respectively (Table S6).

### RNA analyses

Total RNA was extracted from 3 to 4 leaves of 45 days old plants, using Bio-Tri RNA reagent (*Bio-Lab*, Israel) according to the manufacturer’s instructions. DNase (*Ambion*, USA) treatment was performed on RNA samples to remove any residual genomic DNA. For qPCR analyses, 2 µg total RNA was used for first-strand cDNA synthesis using a Maxima first-strand cDNA synthesis kit (*Thermo Scientific*, USA) according to the manufacturer’s instructions. qRT-PCR was performed on the StepOne Plus real-time PCR system (*Applied Biosystems*, USA) using Fast SYBR Green Master Mix according to the manufacturer’s instructions. PCR products were analyzed using StepOne software version 2.2.2 (*Applied Biosystems*, USA). Expression levels were first normalized to a reference gene *TIP41* (SGN-U584254) [24] and relative expression levels were calculated using the 2^−ΔΔCt^ method.

For RNAseq, RNAs were extracted and treated with DNase as above. Total RNA was extracted from 3 to 4 leaves of 45 days old plants and the RNAs of these leaves were combined to sequence the transcriptome of each plant. Three plants (and therefore three biological replicates) were sequenced per genotype. Library preparation and sequencing were performed by *Macrogen* (Korea). On average, 38.4 million single-end 60 bp reads were sequenced per sample on HiSeq 2000 100 cycles run (Table S5). The nf-core/RNAseq (version 3.11.0) pipeline [11] was used for trimming (*TrimGalore*) and aligning (*STAR*) the reads to the SL2.5 version of the tomato genome assembly [46] and to quantify reads matching transcripts (*Salmon*). The differential analysis was then performed with the obtained matrix of raw reads counts using the nf-core differential abundance pipeline (version 1.1.1) which is based on *DESeq2* [32]. RNAseq statistics are listed in Table S5.

### Methylation analyses

To monitor the transient activity of *M.SssI* in tobacco leaves, *Agrobacterium tumefaciens* (strain GV 3101) with respective binary vectors were cultured overnight in LB medium containing the appropriate antibiotics. Cultures were pelleted and suspended in agroinfiltration buffer (10 mM MgCl_2_, 10 mM MES pH 5.6, and 150 µM acetosyringone) to a final O.D. at 600 of 1.0. Bacterial mixtures were then infiltrated into the young leaves of 3-week-old greenhouse-grown *N. benthamiana* plants. Methylation assays were performed after two days. Genomic DNA from leaves was extracted as described previously [38]. Global methylation (5-methyl cytosine [5mC]) was quantified Methylflash using 5-mC DNA ELISA Kit (*ZYMO Research*, USA) according to the manufacturer’s instructions.

To sequence the methylomes, DNA was extracted from 3 to 4 leaves of 45 days old plants with a genomic DNA extraction kit (*Macherey–Nagel*, England). The DNAs of these leaves were combined to sequence the methylome of each plant. Two to three plants (and therefore two to three biological replicates) were sequenced per genotype. Bisulfite treatments, library preparations, and whole-genome sequencings were performed at BGI (China) using HiSeq technology (Illumina), producing 150-bp paired-end reads. Data were trimmed with *Trim_Galore* (*Babraham Bioinformatics*). Reads were aligned to the SL2.5 tomato reference genome assembly [46] with *Bismark* version 0.22.3 (*Babraham Bioinformatics*) and standard options (*Bowtie2*; 1 mismatch allowed). Identical pairs were collapsed. To call Differently Methylated Regions (DMRs) between genotypes, we used the following R packages: *bsseq* version 1.30.0 (Hansen et al., [17] ) and *DSS* version 2.42.0 (Wu et al., [51]). DMRs between the controls and other genotypes were identified considering the variation of each biological replicate. The following minimum thresholds were applied to define a DMR: 30% of difference for CG DMRs, 20% for CHG and 10% for CHH.

TEs were annotated with REPET [12], and the repeat-rich, -intermediate and -poor regions were defined as described [23], using the SL2.50 version of the genome.

Overlap between DMRs and accessible chromatin regions were determined genome-wide using available ATAC-seq (Assay for Transposase-Accessible Chromatin using sequencing) data of meristem-enriched tissue [19]. Peak regions obtained by Hendelman et al. are accessible through GEO Series accession number GSE164297. Methylation and expression correlation were obtained with MethGet [45].

## Conflict of interest

All authors declare that they have no conflicts of interest.

## Supplementary Information

Below is the link to the electronic supplementary material.

Supplementary file1 (PDF 7514 KB)

Supplementary file2 (PDF 365 KB)

## Data Availability

Whole-genome bisulfite single-base resolution sequencing and RNA-seq data described in this study are available from the European Nucleotide Archive database under the accession number PRJEB63616.

## References

[1] Agorio A, Durand S, Fiume E, Brousse C, Gy I, Simon M, Anava S, Rechavi O, Loudet O, Camilleri C et al (2017) An Arabidopsis Natural Epiallele Maintained by a Feed-Forward Silencing Loop between Histone and DNA. PLoS Genet 13:e1006551 28060933 10.1371/journal.pgen.1006551 PMC5257005

[2] Arora H, Singh RK, Sharma S, Sharma N, Panchal A, Das T, Prasad A, Prasad M (2022) DNA methylation dynamics in response to abiotic and pathogen stress in plants. Plant Cell Rep 41:1931–1944 35833989 10.1007/s00299-022-02901-x

[3] Baduel P, Colot V (2021) The epiallelic potential of transposable elements and its evolutionary significance in plants. Philos Trans R Soc Lond B Biol Sci 376:20200123 33866816 10.1098/rstb.2020.0123 PMC8059525

[4] Chaikind B, Ostermeier M (2014) Directed evolution of improved zinc finger methyltransferases. PLoS ONE 9:e96931 24810747 10.1371/journal.pone.0096931 PMC4014571

[5] Choi J, Lyons DB, Zilberman D (2021) Histone H1 prevents non-CG methylation-mediated small RNA biogenesis in Arabidopsis heterochromatin. Elife 10:e72676 34850679 10.7554/eLife.72676 PMC8828055

[6] Cokus SJ, Feng S, Zhang X, Chen Z, Merriman B, Haudenschild CD, Pradhan S, Nelson SF, Pellegrini M, Jacobsen SE (2008) Shotgun bisulphite sequencing of the Arabidopsis genome reveals DNA methylation patterning. Nature 452:215–219 18278030 10.1038/nature06745 PMC2377394

[7] Corem S, Doron-Faigenboim A, Jouffroy O, Maumus F, Arazi T, Bouché N (2018) Redistribution of CHH methylation and small interfering RNAs across the genome of tomato ddm1 mutants. Plant Cell 30:1628–1644 29875274 10.1105/tpc.18.00167 PMC6096599

[8] Cubas P, Vincent C, Coen E (1999) An epigenetic mutation responsible for natural variation in floral symmetry. Nature 401:157–161 10490023 10.1038/43657

[9] Durand S, Bouché N, Perez Strand E, Loudet O, Camilleri C (2012) Rapid establishment of genetic incompatibility through natural epigenetic variation. Curr Biol CB 22:326–331 22285031 10.1016/j.cub.2011.12.054

[10] Eckes P, Rosahl S, Schell J, Willmitzer L (1986) Isolation and characterization of a light-inducible, organ-specific gene from potato and analysis of its expression after tagging and transfer into tobacco and potato shoots. Mol Gen Genet 205:14–22 10.1007/BF02428027

[11] Ewels PA, Peltzer A, Fillinger S, Patel H, Alneberg J, Wilm A, Garcia MU, Di Tommaso P, Nahnsen S (2020) The nf-core framework for community-curated bioinformatics pipelines. Nat Biotechnol 38:276–278 32055031 10.1038/s41587-020-0439-x

[12] Flutre T, Duprat E, Feuillet C, Quesneville H (2011) Considering transposable element diversification in de novo annotation approaches. PLoS ONE 6:e16526 21304975 10.1371/journal.pone.0016526 PMC3031573

[13] Ghoshal B, Picard CL, Vong B, Feng S, Jacobsen SE (2021) CRISPR-based targeting of DNA methylation in Arabidopsis thaliana by a bacterial CG-specific DNA methyltransferase. Proc Natl Acad Sci U S A 118:e2125016118 34074795 10.1073/pnas.2125016118 PMC8201958

[14] Goeldel C, Johannes F (2023) Stochasticity in gene body methylation. Curr Opin Plant Biol 75:102436 37597469 10.1016/j.pbi.2023.102436

[15] Grefen C, Donald N, Hashimoto K, Kudla J, Schumacher K, Blatt MR (2010) A ubiquitin-10 promoter-based vector set for fluorescent protein tagging facilitates temporal stability and native protein distribution in transient and stable expression studies. Plant J Cell Mol Biol 64:355–365 10.1111/j.1365-313X.2010.04322.x 20735773

[16] Groth M, Moissiard G, Wirtz M, Wang H, Garcia-Salinas C, Ramos-Parra PA, Bischof S, Feng S, Cokus SJ, John A et al (2016) MTHFD1 controls DNA methylation in Arabidopsis. Nat Commun 7:11640 27291711 10.1038/ncomms11640 PMC4909953

[17] Hansen KD, Langmead B, Irizarry RA (2012) BSmooth: from whole genome bisulfite sequencing reads to differentially methylated regions. Genome Biol 13(10):R83. 10.1186/gb-2012-13-10-r83 23034175 10.1186/gb-2012-13-10-r83 PMC3491411

[18] Hendelman A, Stav R, Zemach H, Arazi T (2013) The tomato NAC transcription factor SlNAM2 is involved in flower-boundary morphogenesis. J Exp Bot 64:5497–5507 24085581 10.1093/jxb/ert324 PMC3871814

[19] Hendelman A, Zebell S, Rodriguez-Leal D, Dukler N, Robitaille G, Wu X, Kostyun J, Tal L, Wang P, Bartlett ME et al (2021) Conserved pleiotropy of an ancient plant homeobox gene uncovered by cis-regulatory dissection. Cell 184:1724-1739.e16 33667348 10.1016/j.cell.2021.02.001

[20] Hu L, Li N, Xu C, Zhong S, Lin X, Yang J, Zhou T, Yuliang A, Wu Y, Chen Y-R et al (2014) Mutation of a major CG methylase in rice causes genome-wide hypomethylation, dysregulated genome expression, and seedling lethality. Proc Natl Acad Sci U S A 111:10642–10647 25002488 10.1073/pnas.1410761111 PMC4115543

[21] Jacobsen SE, Meyerowitz EM (1997) Hypermethylated SUPERMAN epigenetic alleles in arabidopsis. Science 277:1100–1103 9262479 10.1126/science.277.5329.1100

[22] Johannes F, Porcher E, Teixeira FK, Saliba-Colombani V, Simon M, Agier N, Bulski A, Albuisson J, Heredia F, Audigier P et al (2009) Assessing the Impact of Transgenerational Epigenetic Variation on Complex Traits. PLOS Genet 5:e1000530 19557164 10.1371/journal.pgen.1000530 PMC2696037

[23] Jouffroy O, Saha S, Mueller L, Quesneville H, Maumus F (2016) Comprehensive repeatome annotation reveals strong potential impact of repetitive elements on tomato ripening. BMC Genomics 17:624 27519651 10.1186/s12864-016-2980-z PMC4981986

[24] Lacerda ALM, Fonseca LN, Blawid R, Boiteux LS, Ribeiro SG, Brasileiro ACM (2015) Reference gene selection for qPCR analysis in tomato-bipartite begomovirus interaction and validation in additional tomato-virus pathosystems. PLoS ONE 10:e0136820 26317870 10.1371/journal.pone.0136820 PMC4552598

[25] Law JA, Jacobsen SE (2010) Establishing, maintaining and modifying DNA methylation patterns in plants and animals. Nat Rev Genet 11:204–220 20142834 10.1038/nrg2719 PMC3034103

[26] Lee S, Choi J, Park J, Hong CP, Choi D, Han S, Choi K, Roh T-Y, Hwang D, Hwang I (2023) DDM1-mediated gene body DNA methylation is associated with inducible activation of defense-related genes in arabidopsis. Genome Biol 24:106 37147734 10.1186/s13059-023-02952-7 PMC10161647

[27] Lee SC, Adams DW, Ipsaro JJ, Cahn J, Lynn J, Kim H-S, Berube B, Major V, Calarco JP, LeBlanc C et al (2023) Chromatin remodeling of histone H3 variants by DDM1 underlies epigenetic inheritance of DNA methylation. Cell 24:2023. 10.1016/j.cell.2023.08.001 10.1016/j.cell.2023.08.001 PMC10529913 37643610

[28] Lei Y, Zhang X, Su J, Jeong M, Gundry MC, Huang Y-H, Zhou Y, Li W, Goodell MA (2017) Targeted DNA methylation in vivo using an engineered dCas9-MQ1 fusion protein. Nat Commun 8:16026 28695892 10.1038/ncomms16026 PMC5508226

[29] Lieberman-Lazarovich M, Kaiserli E, Bucher E, Mladenov V (2022) Natural and induced epigenetic variation for crop improvement. Curr Opin Plant Biol 70:102297 36108411 10.1016/j.pbi.2022.102297

[30] Lifschitz E, Eviatar T, Rozman A, Shalit A, Goldshmidt A, Amsellem Z, Alvarez JP, Eshed Y (2006) The tomato FT ortholog triggers systemic signals that regulate growth and flowering and substitute for diverse environmental stimuli. Proc Natl Acad Sci 103:6398–6403 16606827 10.1073/pnas.0601620103 PMC1458889

[31] Liu W, Gallego-Bartolomé J, Zhou Y, Zhong Z, Wang M, Wongpalee SP, Gardiner J, Feng S, Kuo PH, Jacobsen SE (2021) Ectopic targeting of CG DNA methylation in Arabidopsis with the bacterial SssI methyltransferase. Nat Commun 12:3130 34035251 10.1038/s41467-021-23346-y PMC8149686

[32] Love MI, Huber W, Anders S (2014) Moderated estimation of fold change and dispersion for RNA-seq data with DESeq2. Genome Biol 15:550 25516281 10.1186/s13059-014-0550-8 PMC4302049

[33] Lucibelli F, Valoroso MC, Aceto S (2022) Plant DNA methylation: an epigenetic mark in development, environmental interactions, and evolution. Int J Mol Sci 23:8299 35955429 10.3390/ijms23158299 PMC9368846

[34] Lyons DB, Zilberman D (2017) DDM1 and Lsh remodelers allow methylation of DNA wrapped in nucleosomes. Elife 6:e30674 29140247 10.7554/eLife.30674 PMC5728721

[35] Manning K, Tör M, Poole M, Hong Y, Thompson AJ, King GJ, Giovannoni JJ, Seymour GB (2006) A naturally occurring epigenetic mutation in a gene encoding an SBP-box transcription factor inhibits tomato fruit ripening. Nat Genet 38:948–952 16832354 10.1038/ng1841

[36] Meng J, Wang L, Wang J, Zhao X, Cheng J, Yu W, Jin D, Li Q, Gong Z (2018) Methionine Adenosyltransferase4 mediates DNA and histone methylation. Plant Physiol 177:652–670 29572390 10.1104/pp.18.00183 PMC6001336

[37] Moore I, Gälweiler L, Grosskopf D, Schell J, Palme K (1998) A transcription activation system for regulated gene expression in transgenic plants. Proc Natl Acad Sci U S A 95:376–381 9419383 10.1073/pnas.95.1.376 PMC18229

[38] Pavan Kumar BK, Kanakala S, Malathi VG, Gopal P, Usha R (2017) Transcriptomic and proteomic analysis of yellow mosaic diseased soybean. J Plant Biochem Biotechnol 26:224–234 10.1007/s13562-016-0385-3

[39] Reinders J, Wulff BBH, Mirouze M, Marí-Ordóñez A, Dapp M, Rozhon W, Bucher E, Theiler G, Paszkowski J (2009) Compromised stability of DNA methylation and transposon immobilization in mosaic Arabidopsis epigenomes. Genes Dev 23:939–950 19390088 10.1101/gad.524609 PMC2675864

[40] Renbaum P, Abrahamove D, Fainsod A, Wilson GG, Rottem S, Razin A (1990) Cloning, characterization, and expression in Escherichia coli of the gene coding for the CpG DNA methylase from Spiroplasma sp. strain MQ1(M.SssI). Nucleic Acids Res 18:1145–1152 2181400 10.1093/nar/18.5.1145 PMC330428

[41] Shiba H, Kakizaki T, Iwano M, Tarutani Y, Watanabe M, Isogai A, Takayama S (2006) Dominance relationships between self-incompatibility alleles controlled by DNA methylation. Nat Genet 38:297–299 16444272 10.1038/ng1734

[42] Ślaska-Kiss K, Zsibrita N, Koncz M, Albert P, Csábrádi Á, Szentes S, Kiss A (2021) Lowering DNA binding affinity of SssI DNA methyltransferase does not enhance the specificity of targeted DNA methylation in *E. coli*. Sci Rep 11:15226 34315949 10.1038/s41598-021-94528-3 PMC8316445

[43] Stav R, Hendelman A, Buxdorf K, Arazi T (2010) Transgenic expression of tomato bushy stunt virus silencing suppressor P19 via the pOp/LhG4 transactivation system induces viral-like symptoms in tomato. Virus Genes 40:119–129 19859797 10.1007/s11262-009-0415-5

[44] Tan F, Lu Y, Jiang W, Wu T, Zhang R, Zhao Y, Zhou D-X (2018) DDM1 represses noncoding RNA expression and RNA-directed DNA methylation in heterochromatin. Plant Physiol 177:1187–1197 29794169 10.1104/pp.18.00352 PMC6052999

[45] Teng C-S, Wu B-H, Yen M-R, Chen P-Y (2020) MethGET: web-based bioinformatics software for correlating genome-wide DNA methylation and gene expression. BMC Genomics 21:375 32471342 10.1186/s12864-020-6722-x PMC7257144

[46] Tomato Genome Consortium (2012) The tomato genome sequence provides insights into fleshy fruit evolution. Nature 485:635–641 22660326 10.1038/nature11119 PMC3378239

[47] van Blokland R, Ross S, Corrado G, Scollan C, Meyer P (1998) Developmental abnormalities associated with deoxyadenosine methylation in transgenic tobacco. Plant J Cell Mol Biol 15:543–551 10.1046/j.1365-313X.1998.00238.x 9753779

[48] van der Gun BTF, Maluszynska-Hoffman M, Kiss A, Arendzen AJ, Ruiters MHJ, McLaughlin PMJ, Weinhold E, Rots MG (2010) Targeted DNA methylation by a DNA methyltransferase coupled to a triple helix forming oligonucleotide to down-regulate the epithelial cell adhesion molecule. Bioconjug Chem 21:1239–1245 20593890 10.1021/bc1000388 PMC2907751

[49] Weber H, Ziechmann C, Graessmann A (1990) In vitro DNA methylation inhibits gene expression in transgenic tobacco. EMBO J 9:4409–4415 1702383 10.1002/j.1460-2075.1990.tb07891.x PMC552233

[50] Weigel D, Colot V (2012) Epialleles in plant evolution. Genome Biol 13:249 23058244 10.1186/gb-2012-13-10-249 PMC3491404

[51] Wu H, Xu T, Feng H, Chen L, Li B, Yao B, Qin Z, Jin P, Conneely K (2015) Detection of differentially methylated regions from whole-genome bisulfite sequencing data without replicates. Nucleic acids res. 10.1093/nar/gkv715 26184873 10.1093/nar/gkv715 PMC4666378

[52] Xiong T, Meister GE, Workman RE, Kato NC, Spellberg MJ, Turker F, Timp W, Ostermeier M, Novina CD (2017) Targeted DNA methylation in human cells using engineered dCas9-methyltransferases. Sci Rep 7:6732 28751638 10.1038/s41598-017-06757-0 PMC5532369

[53] Xiong T, Rohm D, Workman RE, Roundtree L, Novina CD, Timp W, Ostermeier M (2018) Protein engineering strategies for improving the selective methylation of target CpG sites by a dCas9-directed cytosine methyltransferase in bacteria. PLoS ONE 13:e0209408 30562388 10.1371/journal.pone.0209408 PMC6298699

[54] Xu G-L, Bestor TH (1997) Cytosine methylation targetted to pre-determined sequences. Nat Genet 17:376–378 9398832 10.1038/ng1297-376

[55] Yamazaki T, Hatano Y, Handa T, Kato S, Hoida K, Yamamura R, Fukuyama T, Uematsu T, Kobayashi N, Kimura H et al (2017) Targeted DNA methylation in pericentromeres with genome editing-based artificial DNA methyltransferase. PLoS ONE 12:e0177764 28542388 10.1371/journal.pone.0177764 PMC5436701

[56] Yamazaki T, Hatano Y, Kobayashi N, Yamagata K (2023) Targeted DNA methylation in mouse early embryos. Methods Mol Biol Clifton NJ 2577:243–254 10.1007/978-1-0716-2724-2_17 36173578

[57] Yan X, Ma L, Pang H, Wang P, Liu L, Cheng Y, Cheng J, Guo Y, Li Q (2019) Methionine Synthase1 is involved in chromatin silencing by maintaining DNA and histone methylation1. Plant Physiol 181:249–261 31331996 10.1104/pp.19.00528 PMC6716260

[58] Yifhar T, Pekker I, Peled D, Friedlander G, Pistunov A, Sabban M, Wachsman G, Alvarez JP, Amsellem Z, Eshed Y (2012) Failure of the tomato trans-acting short interfering RNA program to regulate AUXIN RESPONSE FACTOR3 and ARF4 underlies the wiry leaf syndrome. Plant Cell 24:3575–3589 23001036 10.1105/tpc.112.100222 PMC3480288

[59] Zhou M, Palanca AMS, Law JA (2018) Locus-specific control of the de novo DNA methylation pathway in Arabidopsis by the CLASSY family. Nat Genet 50:865–873 29736015 10.1038/s41588-018-0115-y PMC6317521

