# Correction: Genome wide inherited modifications of the tomato epigenome by trans-activated bacterial CG methyltransferase

**DOI:** 10.1007/s00018-024-05387-w

**Published:** 2024-09-10

**Authors:** Bapatla Kesava Pavan Kumar, Sébastien Beaubiat, Chandra Bhan Yadav, Ravit Eshed, Tzahi Arazi, Amir Sherman, Nicolas Bouché

**Affiliations:** 1 Institute of Plant Sciences, Agricultural Research Organization, Volcani Center, Derech Hamacabim 68, Rishon Lezion, Israel; 2 INRAE, AgroParisTech, Institute Jean Pierre Bourgin for Plant Sciences (IJPB), Université Paris-Saclay, Versailles, 78000 France; 3 Molecular Biology, Acrannolife Genomics Private Limited, Chennai, 600035 Tamilnadu India; 4 Department of Genetics, Genomics, and Breeding, NIAB-EMR, East Malling, East Malling, ME19 6BJ UK

**Correction: Cellular and Molecular Life Sciences (2024) 81:222**

10.1007/s00018-024-05255-7

In this article the affiliation details for Author Nicolas Bouché were incorrectly given as ‘Institute of Plant Sciences, Agricultural Research Organization, Volcani Center, Derech Hamacabim 68, Rishon Lezion, Israel’

but should have been

‘INRAE, AgroParisTech, Institute Jean Pierre Bourgin for Plant Sciences (IJPB), Université Paris-Saclay, 78000 Versailles, France’

The original article has been corrected.

