# Supplementary material for: Genome wide inherited modifications of the tomato epigenome by trans-activated bacterial CG methyltransferase
Source: Cell Mol Life Sci. 2024 May 20;81(1):222. doi: 10.1007/s00018-024-05255-7 (PMC11106227; doi:10.1007/s00018-024-05255-7)
Supplement: Supplementary file 2 — Supplementary file2 (PDF 365 KB) [file 18_2024_5255_MOESM2_ESM.pdf]

Sup Table 1. Whole-genome bisulfite sequencing statistics

	Control driver lines				F2-Mss(+)				F2-Mss(-)		F3-Mss(-)	
	**Plant#1**	**Plant#2**	**Plant#3**	**Plant#4**	**Plant#1**	**Plant#2**	**Plant#3**	**Plant#4**	**Plant#1**	**Plant#2**	**Plant#1**	**Plant#2**
Number of clean paired-end reads	120,263,261	120,690,732	174,106,992	174,019,654	120,679,595	120,545,905	103,668,071	117,470,692	118,163,575	117,013,588	167,537,378	174,138,609
Number of mapped paired-end reads	96,773,350	98,764,278	138,591,199	137,439,583	79,997,757	81,876,303	74,533,989	90,921,225	86,014,361	82,464,462	132,537,678	137,723,956
% of Cs methylated in the CG context	77.4%	78.0%	72.3%	74.4%	78.1%	79.4%	77.7%	77.3%	78.7%	75.3%	80.2%	78.4%
% of Cs methylated in the CHG context	55.7%	55.1%	49.6%	50.2%	54.7%	57.1%	52.8%	52.7%	55.0%	52.7%	54.8%	53.9%
% of Cs methylated in the CHH context	9.1%	9.4%	7.4%	6.8%	11.5%	12.4%	11.6%	11.3%	11.9%	10.5%	5.9%	6.8%
Coverage	31.1	31.4	35.3	35.4	25.0	25.5	24.6	29.9	28.3	26.8	34.4	35.7
Bisulfite conversion rates (%)												
CG	99.3%	99.3%	99.1%	99.0%	99.1%	99.1%	98.9%	98.9%	99.0%	99.1%	98.3%	98.9%
CHG	99.3%	99.3%	99.1%	99.0%	99.2%	99.2%	99.0%	99.1%	99.0%	99.2%	98.5%	98.9%
CHH	99.6%	99.6%	99.2%	99.2%	99.5%	99.5%	99.3%	99.3%	99.3%	99.3%	99.2%	99.2%

The number of clean paired-end reads obtained after trimming, the number of mapped paired-end reads, the coverage (after maping and deduplication), and the percentage of cytosines methylated in all contexts are indicated. Bisulfite conversion rates were determined by aligning the reads to the chloroplast sequences of *Solanum lycopersicum* (NCBI reference sequence NC_007898.3). The coverage was obtained using the *bam2nuc* script.

Sup Table 2. Number of DMRs identified in F2 and F3 plants compared to control driver lines

Total count and dimensions of DMRs for each genotype, and distribution of DMRs across regions with low TE density (RP), high TE density (RR), or intermediate TE presence (INT).

			Count/size of DMRs (total)		Distribution of DMRs		
			number	DMR size (bp)	RP	INT	RR
F2-Mss(+) plant #1	CG DMRs	hyper	51795	281	31153	10184	11207
		hypo	2650	176	1269	365	1040
	CHG DMRs	hyper	6076	235	1977	1517	2696
		hypo	5809	206	952	858	4097
	CHH DMRs	hyper	40559	161	21399	10597	9296
		hypo	1788	139	1012	418	390
					RP	INT	RR
F2-Mss(+) plant #2	CG DMRs	hyper	48467	263	29135	9526	10513
		hypo	2770	189	1260	379	1159
	CHG DMRs	hyper	7009	239	2244	1646	3239
		hypo	7650	207	1585	1125	5069
	CHH DMRs	hyper	63586	177	33543	16138	15018
		hypo	914	142	543	206	175
					RP	INT	RR
F2-Mss(+) plant #3	CG DMRs	hyper	48254	277	28871	9483	10612
		hypo	3745	195	2361	517	908
	CHG DMRs	hyper	5187	212	1799	1262	2219
		hypo	8017	197	1784	1168	5187
	CHH DMRs	hyper	52247	165	26939	13201	13027
		hypo	1109	139	652	241	238
F2-Mss(+) plant #4	CG DMRs	hyper	54758	299	32574	10699	12272
		hypo	3877	206	2092	531	1292
	CHG DMRs	hyper	5617	250	1712	1295	2702
		hypo	11506	226	2241	1818	7629
	CHH DMRs	hyper	39870	165	21924	10076	8557
		hypo	1214	166	562	292	381
F2-Mss(-) plant #1	CG DMRs	hyper	12410	186	6867	2338	3382
		hypo	745	176	434	107	208
	CHG DMRs	hyper	7289	226	2170	1538	3695
		hypo	6313	222	1085	928	4404
	CHH DMRs	hyper	60537	169	32850	13954	14756
		hypo	426	180	202	108	122
F2-Mss(-) plant #2	CG DMRs	hyper	11177	180	6490	2176	2668
		hypo	991	158	548	129	325
	CHG DMRs	hyper	6072	238	2160	1401	2607
		hypo	8061	202	1259	1328	5636
	CHH DMRs	hyper	24425	145	13563	5603	5645
		hypo	829	146	455	218	174

			Count/size of DMRs (total)		Distribution of DMRs		
			number	DMR size (bp)	RP	INT	RR
---> F3-Mss(-) plant #1	CG DMRs	hyper	20354	186	13266	3638	3711
		hypo	2760	147	1294	394	1112
	CHG DMRs	hyper	5273	204	2068	1252	2060
		hypo	8253	203	1242	1189	5950
	CHH DMRs	hyper	370	138	218	74	83
		hypo	25173	167	16035	5676	3889
---> F3-Mss(-) plant #2	CG DMRs	hyper	13101	179	7690	2577	3056
		hypo	4921	164	2392	775	1817
	CHG DMRs	hyper	5571	247	1593	1214	2860
		hypo	9441	245	1748	1520	6319
	CHH DMRs	hyper	547	203	240	132	179
		hypo	7957	146	4582	1913	1589

Sup Table 3. RNAseq sequencing statistics

Sample	Total Bases	Read Count	GC (%)	AT (%)	Q20 (%)	Q30 (%)
F2Mss-plant3	4'088'370'718	40'478'918	43.52	56.48	98.15	94.34
F2Mss-plant4	4'796'321'734	47'488'334	43.45	56.55	98.13	94.24
F2Mss-plant5	5'040'808'192	49'908'992	43.49	56.51	98.12	94.23
F2Mss+plant5	4'047'129'590	40'070'590	43.43	56.57	98.11	94.23
F2Mss+plant6	3'898'406'686	38'598'086	43.35	56.65	98.26	94.53
F2Mss+plant7	4'242'682'760	42'006'760	43.32	56.68	98.14	94.33
controlplant5	3'665'476'244	36'291'844	43.45	56.55	98.05	94.05
controlplant6	3'656'051'732	36'198'532	43.42	56.58	98.17	94.35
controlplant7	3'747'230'290	37'101'290	43.13	56.87	98.19	94.40

Sup Table 4: List of upregulated genes between F2-Mss(+) plants and the control driver lines

gene_id	baseMean	log2FoldChange	lfcSE	pvalue	padj
gene:Solyc12g100330.1	341.4361	1.002375	0.4818803	0.000308586	0.0189452
gene:Solyc01g087720.2	487.0926	1.00739	0.26579115	3.32E-06	0.000694626
gene:Solyc06g009680.2	208.2584	1.007704	0.36886821	6.78E-05	0.006863807
gene:Solyc01g108350.2	15.84961	1.027854	1.79148372	0.000785007	0.03560864
gene:Solyc04g071130.1	313.7964	1.03264	0.24052826	6.35E-07	0.000169584
gene:Solyc07g056410.2	202.4333	1.042196	0.68665407	0.000959461	0.04053072
gene:Solyc08g007670.1	151.8148	1.043352	0.45647182	0.000197617	0.01465398
gene:Solyc12g044880.1	533.5657	1.045681	0.38979561	7.98E-05	0.007648742
gene:Solyc06g036060.2	38.32235	1.046843	0.61356785	0.000667177	0.03196017
gene:Solyc08g079860.1	466.6482	1.048283	0.78732436	0.001302062	0.04931359
gene:Solyc09g065390.1	73.01785	1.052935	0.73889426	0.001114049	0.04473135
gene:Solyc05g009650.2	406.5951	1.05953	0.69708383	0.000937266	0.03996381
gene:Solyc04g076140.2	86.75811	1.062984	0.48389114	0.00024076	0.01638166
gene:Solyc06g074500.1	98.88839	1.071848	0.35220561	3.42E-05	0.004099941
gene:Solyc10g012210.1	40.53071	1.080888	0.58969792	0.000508013	0.026408
gene:Solyc02g086580.2	52.65868	1.086803	0.53217283	0.000332335	0.01968054
gene:Solyc02g077710.1	2300.296	1.109625	0.47755572	0.000184744	0.01409663
gene:Solyc09g010690.2	964.6848	1.110837	0.58799417	0.000447068	0.02400077
gene:Solyc07g055460.2	29.81812	1.119066	1.67540221	0.001010404	0.04216264
gene:Solyc09g074230.2	529.2474	1.119497	0.65668913	0.000630105	0.0308407
gene:Solyc02g091670.1	115.4466	1.136339	0.54692842	0.00030483	0.01876856
gene:Solyc02g076800.1	164.7425	1.143546	0.37559961	3.75E-05	0.004442152
gene:Solyc01g086660.2	84.40306	1.146791	0.8021526	0.000963707	0.04053072
gene:Solyc03g007870.2	1194.59	1.148332	0.64634513	0.000538232	0.02764262
gene:Solyc03g005570.2	293.5073	1.149689	0.88295574	0.001134876	0.04529992
gene:Solyc07g055710.2	372.761	1.15314	0.76270598	0.000842896	0.03751764
gene:Solyc01g095630.2	2118.204	1.155517	0.48717907	0.000167579	0.01301939
gene:Solyc08g080650.1	5256.858	1.158938	1.06438095	0.001330622	0.04988775
gene:Solyc06g051260.2	133.0599	1.159557	0.60357857	0.000407928	0.02276712
gene:Solyc10g076510.1	179.8359	1.169531	0.92988801	0.001146458	0.04561281
gene:Solyc12g005940.1	98.75981	1.170392	1.08980572	0.001304104	0.04931359
gene:Solyc07g055080.2	245.5933	1.171982	0.46042724	0.000116979	0.01001291
gene:Solyc05g052680.1	107.2752	1.176918	1.35628441	0.001260267	0.04861108
gene:Solyc11g008440.1	212.1903	1.181095	0.32561025	8.81E-06	0.001471248
gene:Solyc07g045100.1	341.8363	1.183055	0.61879414	0.000408135	0.02276712
gene:Solyc12g009300.1	168.1554	1.190102	0.474398	0.000126605	0.01064927
gene:Solyc11g008530.1	437.3991	1.199581	0.46371065	0.000107759	0.009320899
gene:Solyc07g049550.2	256.5415	1.200334	0.94766625	0.001068924	0.04358315
gene:Solyc12g010440.1	132.0414	1.20122	0.58046983	0.000300546	0.01873941
gene:Solyc09g092760.2	109.6661	1.205579	0.77625022	0.00073432	0.03366682
gene:Solyc03g114500.2	460.044	1.205601	0.82919938	0.000847666	0.03765154
gene:Solyc06g066770.1	167.6796	1.207722	0.56881087	0.000270475	0.01745832
gene:Solyc07g007170.2	463.0431	1.214159	0.5380335	0.000208826	0.01522717
gene:Solyc11g066330.1	227.3807	1.214358	0.30589933	3.43E-06	0.000711972
gene:Solyc04g007760.2	15.35227	1.220503	1.91289987	0.000585714	0.02951363

gene:Solyc05g008540.2	20.38506	1.222135	0.56349274	0.000246239	0.01664843
gene:Solyc06g066840.2	94.83042	1.223128	1.09543284	0.001136473	0.04529992
gene:Solyc03g097610.2	646.6394	1.225107	0.5667608	0.000249264	0.01674694
gene:Solyc08g007570.2	76.78254	1.22515	0.65239435	0.000418757	0.02305863
gene:Solyc12g027540.1	11.45504	1.231962	0.9908425	0.001024353	0.04243358
gene:Solyc01g105300.2	21.06099	1.23937	1.16653304	0.001118022	0.04473135
gene:Solyc08g077210.2	855.6702	1.24551	0.32369256	5.22E-06	0.000979025
gene:Solyc03g083480.2	623.7844	1.255706	0.9709417	0.000933751	0.03992278
gene:Solyc05g015850.2	287.3131	1.260777	1.43585965	0.001034351	0.04265034
gene:Solyc08g060920.2	293.3167	1.261932	0.55326075	0.00019536	0.01465398
gene:Solyc03g117590.2	2543.916	1.264724	0.62833047	0.000318551	0.01924815
gene:Solyc01g005500.2	13.18391	1.273688	1.51810137	0.000969128	0.04067864
gene:Solyc12g055710.1	32.62982	1.282804	0.86243498	0.000713504	0.03328387
gene:Solyc04g080520.1	95.69969	1.284858	0.38890759	2.29E-05	0.003151328
gene:Solyc07g065320.2	2170.088	1.287802	0.43018932	4.63E-05	0.005180963
gene:Solyc06g074360.2	12.70634	1.292278	1.07726303	0.000934303	0.03992278
gene:Solyc06g071580.2	193.2214	1.295309	0.37245076	1.50E-05	0.002247309
gene:Solyc02g081850.2	575.4082	1.300542	0.54441358	0.000156435	0.01247102
gene:Solyc04g078540.2	636.6608	1.301683	0.22104942	9.54E-10	6.79E-07
gene:Solyc04g064630.2	6.896174	1.301712	1.87464099	0.000621862	0.03061309
gene:Solyc05g051780.2	151.8962	1.31247	0.25932937	6.65E-08	2.78E-05
gene:Solyc01g079140.2	51.26915	1.318385	0.84560718	0.000612472	0.03043129
gene:Solyc08g080190.2	120.1219	1.334821	0.72625536	0.000396872	0.02249116
gene:Solyc09g011990.1	78.97583	1.336079	0.69382087	0.000344133	0.02025456
gene:Solyc11g012930.1	825.6921	1.336799	0.76497945	0.000455228	0.02425425
gene:Solyc09g007140.2	82.8581	1.364632	0.76616348	0.000416457	0.02304304
gene:Solyc05g008210.2	108.2228	1.369643	0.96923437	0.000666795	0.03196017
gene:Solyc01g103390.2	44.60026	1.372753	0.51656807	9.09E-05	0.008225197
gene:Solyc05g009230.1	145.3765	1.38037	0.51321794	8.51E-05	0.007905729
gene:Solyc12g009480.1	183.9734	1.394683	0.32917773	1.81E-06	0.000412214
gene:Solyc09g082220.1	15.71283	1.397359	0.85107116	0.000483101	0.02542231
gene:Solyc02g082920.2	19385.24	1.397654	1.10340217	0.000720151	0.0334619
gene:Solyc10g008120.2	233.3956	1.403352	0.71254265	0.000296039	0.01860034
gene:Solyc08g068610.2	211.3496	1.42303	1.13990461	0.000693915	0.03263811
gene:Solyc09g073040.2	18.85446	1.428596	1.68036086	0.000682309	0.03243501
gene:Solyc01g010060.2	155.1984	1.436939	0.97941012	0.000557016	0.02847043
gene:Solyc02g067690.2	501.2118	1.451606	0.22245113	1.81E-11	2.76E-08
gene:Solyc02g069690.1	160.5459	1.46089	0.48603161	4.39E-05	0.004963246
gene:Solyc09g010360.2	364.2092	1.469498	0.72538704	0.000249231	0.01674694
gene:Solyc04g077990.2	16.19825	1.500657	1.31125284	0.000630816	0.0308407
gene:Solyc10g018120.1	85.7628	1.508703	0.94795874	0.000427108	0.02345802
gene:Solyc09g018230.1	198.9432	1.515678	0.24223821	9.20E-11	1.03E-07
gene:Solyc01g057270.2	20.15451	1.522087	0.58997779	9.37E-05	0.008344386
gene:Solyc07g018200.1	56.24541	1.522373	1.42953376	0.000618312	0.03057926
gene:Solyc05g056500.1	77.21236	1.528427	0.76274544	0.000237804	0.01628425
gene:Solyc05g007770.2	585.809	1.530573	0.87012985	0.000334328	0.01973183
gene:Solyc11g007200.1	1229.873	1.536664	0.38837435	4.31E-06	0.000845389
gene:Solyc09g065120.2	5061.124	1.543965	0.52920703	4.93E-05	0.00537913

gene:Solyc08g068850.2	26.91896	1.549709	0.75746135	0.000216757	0.015426
gene:Solyc12g008680.1	18.61979	1.553526	0.70127388	0.00016626	0.012964
gene:Solyc02g077770.2	119.9318	1.560893	0.43848574	1.22E-05	0.001893425
gene:Solyc09g014990.2	778.9153	1.571364	0.70692211	0.000160509	0.01274822
gene:Solyc10g007290.2	460.2584	1.574436	0.29311542	1.36E-08	7.45E-06
gene:Solyc03g116480.1	106.3698	1.589383	0.85630177	0.000267596	0.01741634
gene:Solyc08g014570.2	421.5918	1.600513	0.55484646	4.98E-05	0.005401335
gene:Solyc10g086280.1	848.9063	1.623668	0.39971653	2.95E-06	0.000637694
gene:Solyc01g112000.2	820.8798	1.62985	0.22551068	9.91E-14	3.53E-10
gene:Solyc03g083590.2	50.06731	1.646239	0.36408	5.88E-07	0.000159669
gene:Solyc12g088530.1	60.91296	1.650331	0.53855814	3.34E-05	0.00403282
gene:Solyc09g009690.2	996.3906	1.656933	0.36285253	4.87E-07	0.000136802
gene:Solyc11g012910.1	32.61335	1.660853	0.45117947	8.30E-06	0.00141802
gene:Solyc10g076610.1	33.70978	1.672838	1.67916702	0.000462983	0.02454499
gene:Solyc04g071800.2	839.0494	1.676861	0.25450766	7.74E-12	1.51E-08
gene:Solyc05g056380.2	36.48751	1.677972	1.38332196	0.000441447	0.02381693
gene:Solyc09g011610.2	14.99681	1.679391	1.17646975	0.000375809	0.02159213
gene:Solyc11g012690.1	64.18936	1.6794	0.3282531	4.12E-08	1.83E-05
gene:Solyc09g082230.1	219.4821	1.708112	0.58959238	4.30E-05	0.004907621
gene:Solyc07g055690.1	36.04863	1.709204	1.00147283	0.000266047	0.01738258
gene:Solyc01g110800.2	7.254863	1.730346	2.16068976	0.000285541	0.01815651
gene:Solyc05g009610.1	410.0513	1.745885	0.62686612	5.01E-05	0.005402581
gene:Solyc00g217960.1	64.89946	1.749212	0.49991927	1.17E-05	0.001832244
gene:Solyc01g010230.2	321.1787	1.750238	0.17726569	8.43E-24	1.80E-19
gene:Solyc08g079730.1	37.88215	1.771387	0.99977061	0.000222223	0.01561774
gene:Solyc06g068500.2	2351.003	1.789686	0.68040782	6.15E-05	0.006379098
gene:Solyc06g068940.2	9.63001	1.829029	1.4346218	0.000330555	0.01968054
gene:Solyc12g098780.1	18.83397	1.837907	1.67548743	0.000350273	0.02050294
gene:Solyc10g079420.1	1380.912	1.838235	0.38564575	1.50E-07	5.32E-05
gene:Solyc01g100920.2	91.09602	1.839086	0.80787896	0.000100173	0.00884378
gene:Solyc02g078160.2	296.5793	1.84651	0.64054871	3.64E-05	0.004338869
gene:Solyc01g100200.2	1307.398	1.85263	0.401872	2.84E-07	8.78E-05
gene:Solyc08g067760.2	13.22778	1.863546	1.0224399	0.000180123	0.01379326
gene:Solyc11g072930.1	23.76639	1.895501	1.3514022	0.000269965	0.01745832
gene:Solyc11g066250.1	496.1279	1.907025	0.31213889	8.88E-11	1.03E-07
gene:Solyc09g011540.2	35.50011	1.908864	1.47204068	0.000287329	0.01819334
gene:Solyc04g040120.1	110.7731	1.927784	1.74013236	0.000301821	0.01874537
gene:Solyc03g093140.2	301.6011	1.981107	0.35661555	1.85E-09	1.27E-06
gene:Solyc05g050130.2	6094.064	1.999872	0.67691532	2.50E-05	0.003377585
gene:Solyc02g086960.2	7.881882	2.032759	2.02891123	0.000240437	0.01638166
gene:Solyc05g046290.2	29.87052	2.041611	0.89453909	7.26E-05	0.007198936
gene:Solyc03g111690.2	483.0454	2.043272	0.29996966	5.63E-13	1.50E-09
gene:Solyc10g055810.1	9600.3	2.046617	0.54797265	3.83E-06	0.000779413
gene:Solyc03g095770.2	1755.069	2.048809	0.4794499	6.63E-07	0.000174816
gene:Solyc11g005350.1	413.5142	2.055888	0.63257788	1.25E-05	0.001913941
gene:Solyc01g109680.2	66.46559	2.086773	0.37065419	8.52E-10	6.28E-07
gene:Solyc01g106510.2	19.69522	2.090219	0.88885342	6.07E-05	0.006345879
gene:Solyc10g007280.2	880.8283	2.091373	0.96813043	8.07E-05	0.007692915

gene:Solyc02g094400.2	339.8682	2.104729	0.3892164	2.78E-09	1.80E-06
gene:Solyc03g006490.2	1459.968	2.160998	0.85053606	4.02E-05	0.004668452
gene:Solyc03g034390.1	16.40959	2.186989	1.75996272	0.000197009	0.01465398
gene:Solyc02g068190.1	28.59458	2.307862	0.44968318	6.06E-09	3.50E-06
gene:Solyc02g082740.1	287.9718	2.323964	0.70805345	7.11E-06	0.001254802
gene:Solyc08g080620.1	2826.756	2.331035	2.31728725	0.00013626	0.01126646
gene:Solyc12g094600.1	7.021757	2.341036	1.77088295	0.000153143	0.01227737
gene:Solyc12g057090.1	84.08966	2.344528	0.52010821	1.11E-07	4.16E-05
gene:Solyc03g005460.2	95.74972	2.362291	0.33974276	6.39E-14	3.41E-10
gene:Solyc07g005380.2	45.23095	2.39995	1.69941298	0.000133254	0.01112102
gene:Solyc07g042430.1	47.65533	2.412293	1.77849807	0.000136579	0.01126646
gene:Solyc04g051490.2	83.53521	2.432591	0.33168643	3.06E-15	3.27E-11
gene:Solyc09g098510.2	1360.506	2.438589	0.54958549	1.16E-07	4.26E-05
gene:Solyc01g091490.2	77.30206	2.456278	0.58081252	2.65E-07	8.33E-05
gene:Solyc03g036470.1	18.27031	2.456606	1.63143677	0.000114367	0.00985263
gene:Solyc01g080900.2	58.95549	2.489741	0.38435811	1.06E-12	2.51E-09
gene:Solyc00g026160.2	9901.052	2.50507	0.42845775	5.51E-11	7.85E-08
gene:Solyc04g007000.1	596.096	2.527451	0.41909107	1.66E-11	2.72E-08
gene:Solyc06g033860.1	92.66595	2.742751	1.2290513	3.14E-05	0.003853203
gene:Solyc11g010670.1	34.02066	2.759153	0.58371991	1.31E-08	7.39E-06
gene:Solyc03g025460.2	22.00318	2.775662	1.00597047	1.16E-05	0.001832244
gene:Solyc12g056920.1	21.49372	2.957757	0.75022785	3.09E-07	9.42E-05
gene:Solyc01g094380.2	36.74294	3.013659	1.13910634	1.14E-05	0.001817194
gene:Solyc05g054010.2	51.64497	3.052154	0.90586896	1.94E-06	0.000435922
gene:Solyc02g082910.2	51.19828	3.073165	1.47538262	2.88E-05	0.003726344
gene:Solyc08g016270.1	44.90481	3.125969	0.79410232	2.60E-07	8.29E-05
gene:Solyc01g010910.1	18.07336	3.160761	1.20894922	1.07E-05	0.001724265
gene:Solyc03g006700.2	782.3427	3.306819	1.49751104	1.94E-05	0.002724976
gene:Solyc01g099660.2	49.42795	3.311485	0.6770584	2.75E-09	1.80E-06
gene:Solyc05g056400.2	61.54536	3.539661	1.61147338	1.63E-05	0.002376062
gene:Solyc11g069880.1	28.69746	3.637082	1.90098585	2.17E-05	0.003007037
gene:Solyc02g078150.2	384.3341	3.731784	0.94871705	1.99E-07	6.65E-05
gene:Solyc02g086970.2	2861.048	3.970768	0.73529094	2.24E-10	1.99E-07
gene:Solyc03g020030.2	76.41529	3.985491	1.4477253	4.70E-06	0.000888036
gene:Solyc08g080590.2	256.654	4.000091	0.75717281	4.32E-10	3.69E-07
gene:Solyc09g007940.2	55.22112	4.079544	1.59316999	6.20E-06	0.001112954
gene:Solyc11g005480.1	117.7695	4.694104	1.37953011	8.73E-07	0.000219542
gene:Solyc08g078180.1	80.80045	4.732774	1.53128823	1.63E-06	0.000373449
gene:Solyc04g071070.2	849.151	4.935137	0.87840782	1.76E-10	1.71E-07
gene:Solyc00g020840.2	199.1032	6.093411	1.68323007	1.95E-07	6.62E-05

Sup Table 5: List of downregulated genes between F2-Mss(+) plants and the control driver lines

gene_id	baseMean	log2FoldChange	lfcSE	pvalue	padj
gene:Solyc07g042230.1	34.93491	-6.170117	1.71736578	1.86E-07	6.52E-05
gene:Solyc08g075210.1	194.0637	-5.90794	0.85262144	9.54E-14	3.53E-10
gene:Solyc01g095140.2	1353.009	-5.44282	0.95959276	1.74E-10	1.71E-07
gene:Solyc06g072670.2	32.37331	-5.327921	1.85465578	1.39E-06	0.000330669
gene:Solyc10g086690.1	51.98079	-5.256407	1.44421978	3.95E-07	0.000112525
gene:Solyc05g015800.2	57.24598	-5.005243	1.3479308	3.72E-07	0.00010736
gene:Solyc06g083900.2	181.871	-4.72494	1.19665357	2.03E-07	6.66E-05
gene:Solyc06g062370.2	1554.037	-4.657158	0.76021972	7.76E-12	1.51E-08
gene:Solyc11g071480.1	16.60675	-4.554241	2.00658155	6.08E-06	0.001100915
gene:Solyc08g083500.1	36.72113	-4.39689	0.78365129	1.10E-10	1.17E-07
gene:Solyc12g010970.1	49.29395	-4.15693	1.19574361	8.43E-07	0.000214475
gene:Solyc01g091170.2	2482.514	-3.790216	0.72652859	5.25E-10	4.31E-07
gene:Solyc04g012050.2	110.996	-3.690598	1.35666137	5.95E-06	0.001087368
gene:Solyc09g089540.2	37.84866	-3.574106	2.3016802	2.93E-05	0.003745812
gene:Solyc04g008330.1	53.10961	-3.540656	1.20124036	4.03E-06	0.00080459
gene:Solyc06g084190.2	9.140453	-3.469278	1.96709973	2.97E-05	0.003776223
gene:Solyc09g065620.2	34.17245	-3.38018	1.00051994	1.45E-06	0.000341522
gene:Solyc03g098790.1	449.8185	-3.374082	1.27688846	8.55E-06	0.001438908
gene:Solyc12g096780.1	57.57978	-3.358162	0.98173301	1.30E-06	0.000312296
gene:Solyc10g079980.1	21.25006	-3.346901	1.44353916	1.57E-05	0.00229692
gene:Solyc01g108800.2	76.63693	-3.307857	0.51808027	4.07E-13	1.24E-09
gene:Solyc02g085490.1	18.4365	-3.220289	2.23106234	4.71E-05	0.005234732
gene:Solyc01g088230.2	7.200877	-3.186301	2.29229457	4.91E-05	0.005377892
gene:Solyc08g065430.2	641.8166	-3.150957	0.81843731	3.54E-07	0.000105126
gene:Solyc03g007790.2	37.31878	-3.096947	0.82054857	4.94E-07	0.000137095
gene:Solyc08g066660.1	24.77879	-3.012244	1.08056481	8.53E-06	0.001438908
gene:Solyc10g080690.1	133.16	-2.99801	1.39352231	2.77E-05	0.00361343
gene:Solyc07g006570.2	875.1292	-2.97051	0.945514	3.91E-06	0.000788914
gene:Solyc02g069490.2	3232.822	-2.939988	1.02004824	7.47E-06	0.001296961
gene:Solyc05g055650.2	55.92656	-2.927326	0.65223558	3.04E-08	1.51E-05
gene:Solyc10g086500.1	571.528	-2.902399	0.68792477	1.07E-07	4.16E-05
gene:Solyc01g090560.2	174.8458	-2.889932	0.6441675	3.26E-08	1.58E-05
gene:Solyc08g007830.1	40.48235	-2.888906	1.94630244	7.00E-05	0.007034428
gene:Solyc11g005240.1	7.826506	-2.879417	2.28392072	7.31E-05	0.007198936
gene:Solyc03g111290.1	142.7603	-2.776976	1.5867427	6.08E-05	0.006345879
gene:Solyc09g007150.2	302.6652	-2.709059	0.55063647	5.55E-09	3.29E-06
gene:Solyc04g008370.1	87.31458	-2.683646	0.82396017	4.17E-06	0.000824246
gene:Solyc01g005870.1	1224.009	-2.652314	0.90044144	9.11E-06	0.001497445
gene:Solyc12g010960.1	42.58742	-2.607082	1.17866945	3.76E-05	0.004442152
gene:Solyc07g062930.2	76.65737	-2.569997	0.47787313	6.87E-10	5.25E-07
gene:Solyc04g054740.2	20392.61	-2.565751	1.44866596	7.45E-05	0.007299861
gene:Solyc03g013160.2	1987.563	-2.546699	0.84390582	9.02E-06	0.001494141
gene:Solyc09g083440.2	4933.715	-2.535422	0.93685231	1.74E-05	0.002490019
gene:Solyc09g008060.2	439.3945	-2.491105	0.9211573	1.85E-05	0.002620155
gene:Solyc04g051360.2	85.12711	-2.404338	2.17631787	0.000139054	0.0114075

gene:Solyc01g097520.2	5618.239	-2.344192	0.66571516	3.72E-06	0.000765057
gene:Solyc12g096770.1	782.1465	-2.317902	1.22642728	8.58E-05	0.007940108
gene:Solyc05g005960.2	1616.628	-2.316231	0.51141111	1.10E-07	4.16E-05
gene:Solyc08g083370.2	114.5749	-2.281199	0.87588198	3.01E-05	0.003799451
gene:Solyc10g079640.1	769.2232	-2.26424	0.47507295	4.35E-08	1.90E-05
gene:Solyc10g005360.2	80.78268	-2.263403	0.55160693	7.10E-07	0.00018506
gene:Solyc07g064600.2	2059.256	-2.258538	1.04330933	6.30E-05	0.006467985
gene:Solyc06g050520.1	5.943626	-2.256092	1.84934732	0.000179883	0.01379326
gene:Solyc01g090330.1	16.8826	-2.252508	2.20775673	0.00016362	0.01292779
gene:Solyc00g052940.2	121.0713	-2.250968	1.02579118	6.09E-05	0.006345879
gene:Solyc12g010980.1	1190.76	-2.238213	1.41717143	0.000139357	0.0114075
gene:Solyc03g078780.1	41.4898	-2.237408	0.51973782	3.54E-07	0.000105126
gene:Solyc08g005320.2	18.17883	-2.23627	0.55275182	9.35E-07	0.000232206
gene:Solyc12g010020.1	708.4305	-2.227838	1.00328184	6.05E-05	0.006345879
gene:Solyc02g087110.2	286.0134	-2.227352	0.46084481	3.55E-08	1.65E-05
gene:Solyc02g063000.2	10.02344	-2.202973	1.73855027	0.000190461	0.0143788
gene:Solyc06g071750.2	10.57135	-2.198646	1.49417051	0.000164027	0.01292779
gene:Solyc09g089510.2	2232.381	-2.193412	1.11927183	9.23E-05	0.0082855
gene:Solyc11g066670.1	657.9239	-2.191161	1.24070059	0.000119195	0.01010558
gene:Solyc02g084330.2	7.498982	-2.148842	1.90677004	0.000212513	0.015339
gene:Solyc07g055840.2	95.96163	-2.140034	0.43308938	2.78E-08	1.42E-05
gene:Solyc08g076970.2	11098.91	-2.133693	0.28724661	4.70E-15	3.35E-11
gene:Solyc01g096370.2	1089.548	-2.12586	0.51953539	1.05E-06	0.000257654
gene:Solyc02g085430.2	34.02353	-2.094956	0.57596824	4.53E-06	0.000864531
gene:Solyc06g074420.1	148.8634	-2.088024	1.61175426	0.000220465	0.01554532
gene:Solyc09g084490.2	1358.915	-2.082077	1.75752827	0.000234313	0.01620099
gene:Solyc03g098780.1	4359.588	-2.077378	0.94686365	7.84E-05	0.007580676
gene:Solyc02g094040.2	8.864983	-2.07238	1.17264347	0.000140568	0.01141916
gene:Solyc02g084770.2	35.08538	-2.067884	0.4925938	8.33E-07	0.000214347
gene:Solyc08g007210.2	67.40899	-2.058926	0.50374974	1.25E-06	0.000304682
gene:Solyc02g085730.2	3922.956	-2.044465	0.63937308	1.42E-05	0.002139773
gene:Solyc03g115370.2	563.4929	-2.006413	0.52419681	3.20E-06	0.000675945
gene:Solyc06g054240.2	21.80232	-2.004694	0.7763801	4.79E-05	0.005277982
gene:Solyc01g090340.2	483.8723	-1.998535	1.7327207	0.000268195	0.01741634
gene:Solyc11g022530.1	24.22839	-1.933577	0.62346267	2.09E-05	0.002919409
gene:Solyc06g007120.2	1209.011	-1.924475	0.55707209	9.88E-06	0.001611027
gene:Solyc07g054620.2	36.83931	-1.894827	0.3875711	7.44E-08	3.00E-05
gene:Solyc06g051940.2	408.8909	-1.886243	1.09334927	0.000195673	0.01465398
gene:Solyc02g086650.2	744.5886	-1.878972	1.53635534	0.000314826	0.01916311
gene:Solyc02g072440.2	21.91916	-1.865463	1.46385398	0.000312067	0.01907616
gene:Solyc01g105650.2	78.95627	-1.862317	1.81150978	0.000332538	0.01968054
gene:Solyc02g090340.2	263.6189	-1.845182	0.69588633	5.47E-05	0.005868648
gene:Solyc06g066540.1	151.8963	-1.828853	1.19398894	0.000265318	0.01738258
gene:Solyc02g091020.1	28.11219	-1.818768	0.76801613	8.99E-05	0.008207358
gene:Solyc03g093700.1	18.21884	-1.814836	0.90538163	0.000153431	0.01227737
gene:Solyc06g084100.2	5.590824	-1.806488	1.80863244	0.000362318	0.02092139
gene:Solyc03g098300.1	13.15242	-1.770525	1.57470481	0.000390544	0.02219144
gene:Solyc04g079730.1	690.4547	-1.761608	1.32744964	0.000355604	0.02064533

gene:Solyc03g078790.1	10.57402	-1.758173	1.21170228	0.000322023	0.01932592
gene:Solyc08g005350.2	195.813	-1.752784	0.28104052	6.26E-11	8.36E-08
gene:Solyc12g035710.1	78.77038	-1.730006	0.42626068	2.63E-06	0.000585518
gene:Solyc05g005950.2	3115.786	-1.72578	0.56493671	3.11E-05	0.003853203
gene:Solyc01g016370.1	40.52494	-1.722699	1.28618068	0.000375955	0.02159213
gene:Solyc07g053890.2	250.8708	-1.720197	0.66516968	7.31E-05	0.007198936
gene:Solyc06g063330.2	97.85574	-1.70728	0.43789059	4.37E-06	0.000848095
gene:Solyc12g098900.1	59.09244	-1.704523	1.27057999	0.000387087	0.02211262
gene:Solyc06g083040.2	5316.582	-1.703828	0.77841519	0.000140135	0.01141916
gene:Solyc02g068140.2	730.4193	-1.690325	0.59161469	4.73E-05	0.005234732
gene:Solyc03g062900.2	70.47614	-1.689829	0.32986617	3.91E-08	1.78E-05
gene:Solyc01g097390.2	77.00749	-1.68963	0.45386362	7.22E-06	0.001263709
gene:Solyc02g065240.2	86.06921	-1.682544	0.29977824	3.04E-09	1.91E-06
gene:Solyc02g071720.2	67.53885	-1.680936	1.02834316	0.00030315	0.01876856
gene:Solyc03g034460.2	33.51938	-1.678023	0.54819611	3.26E-05	0.003956295
gene:Solyc01g108880.2	92.91766	-1.676886	0.41598086	3.07E-06	0.000655492
gene:Solyc05g005920.2	897.7427	-1.662424	0.31593771	2.06E-08	1.07E-05
gene:Solyc12g088230.1	550.2587	-1.650483	0.74876847	0.000148059	0.01198211
gene:Solyc08g083230.1	61.43343	-1.648519	0.55730911	4.12E-05	0.004753252
gene:Solyc00g020540.1	10.65483	-1.630986	1.85337004	0.000454456	0.02425425
gene:Solyc02g070890.2	328.3922	-1.626456	1.24983618	0.000457016	0.02428894
gene:Solyc02g084350.2	63.76807	-1.626209	0.83664511	0.000224136	0.0156715
gene:Solyc01g087590.2	489.0451	-1.623428	1.40594573	0.000499722	0.02604037
gene:Solyc02g077420.2	658.1134	-1.616436	0.7243521	0.00014898	0.01201117
gene:Solyc06g068960.1	1095.099	-1.611181	0.86382837	0.000255422	0.01696827
gene:Solyc07g025170.2	75.0152	-1.605872	0.51460797	3.12E-05	0.003853203
gene:Solyc05g005930.1	433.7338	-1.60049	0.30930044	3.54E-08	1.65E-05
gene:Solyc03g013250.2	56.9294	-1.590405	0.83863965	0.000252897	0.01693774
gene:Solyc11g010170.1	1413.435	-1.573862	0.59960599	8.21E-05	0.007699371
gene:Solyc05g051460.2	595.9477	-1.572181	0.56943271	6.45E-05	0.006588507
gene:Solyc09g008640.1	362.3842	-1.565259	0.32085032	1.47E-07	5.32E-05
gene:Solyc10g008410.1	852.1116	-1.557852	1.06022605	0.000447101	0.02400077
gene:Solyc05g052470.2	660.661	-1.552823	0.4343492	1.18E-05	0.001846871
gene:Solyc03g116100.2	535.4516	-1.552677	0.60153418	9.06E-05	0.008225197
gene:Solyc05g005980.2	497.5385	-1.551079	0.95636409	0.000383387	0.02195995
gene:Solyc07g025160.2	238.104	-1.543733	0.30642177	7.30E-08	3.00E-05
gene:Solyc06g051060.2	27.05739	-1.541856	0.51764356	4.39E-05	0.004963246
gene:Solyc02g080120.1	185.6092	-1.536173	1.08787907	0.000491068	0.02571484
gene:Solyc12g057030.1	67.59374	-1.51826	0.50341208	4.15E-05	0.004768273
gene:Solyc08g078090.1	407.4595	-1.515431	0.55411485	7.13E-05	0.007121381
gene:Solyc03g116590.2	824.9216	-1.508503	0.66849831	0.000164585	0.01292779
gene:Solyc02g088790.2	346.7622	-1.505312	0.2665533	3.40E-09	2.08E-06
gene:Solyc07g005330.2	2404.137	-1.504137	0.91922266	0.000408011	0.02276712
gene:Solyc07g062700.2	14671.38	-1.504018	0.99702543	0.000472632	0.0249328
gene:Solyc01g110460.2	262.3901	-1.501258	0.23850082	7.43E-11	9.33E-08
gene:Solyc06g072430.1	76.1671	-1.492915	0.90141975	0.000405665	0.02276712
gene:Solyc12g042600.1	1065.715	-1.491852	0.46070325	2.66E-05	0.003569254
gene:Solyc10g081840.1	106.983	-1.483154	0.89257588	0.000409453	0.02277233

gene:Solyc01g096730.2	276.3153	-1.482352	0.5550143	8.25E-05	0.007699371
gene:Solyc06g068620.2	534.5748	-1.46169	0.8133531	0.00035347	0.02063358
gene:Solyc01g080910.2	645.2665	-1.460105	0.63937881	0.000166177	0.012964
gene:Solyc01g091320.2	961.4048	-1.45412	1.275367	0.000692049	0.03263811
gene:Solyc11g073080.1	35.05266	-1.435503	0.6605298	0.000205077	0.01505663
gene:Solyc03g006900.1	12.25034	-1.435003	1.218401	0.000706639	0.03303577
gene:Solyc12g036150.1	113.1738	-1.430806	0.28844358	1.11E-07	4.16E-05
gene:Solyc02g089010.1	39.41249	-1.427912	0.80963708	0.000389841	0.02219144
gene:Solyc07g007250.2	4204.205	-1.409298	1.02414845	0.000642343	0.03126436
gene:Solyc10g081570.1	1135.908	-1.388034	1.02281316	0.000680699	0.03243501
gene:Solyc11g006230.1	99.81088	-1.38697	0.80030097	0.00043278	0.02358759
gene:Solyc11g011570.1	103.9231	-1.382722	0.39680746	1.54E-05	0.002280146
gene:Solyc00g009130.2	47.70389	-1.375958	0.36025447	6.41E-06	0.001140472
gene:Solyc01g081160.2	11.62794	-1.374455	1.00636273	0.000692833	0.03263811
gene:Solyc01g094010.2	839.5902	-1.370081	0.70345595	0.000319172	0.01924815
gene:Solyc01g107800.2	125.3672	-1.350678	0.75363229	0.000417396	0.02304304
gene:Solyc11g005430.1	31.32725	-1.347342	1.16329178	0.000852851	0.03780322
gene:Solyc02g071380.2	109.5405	-1.344627	0.65547318	0.00027665	0.01774965
gene:Solyc06g035940.2	265.7163	-1.339991	1.47082968	0.000879283	0.03873376
gene:Solyc12g036140.1	226.5248	-1.33899	0.24910485	1.52E-08	8.11E-06
gene:Solyc01g099290.2	77.59252	-1.337573	0.35952548	8.23E-06	0.00141802
gene:Solyc06g065910.2	8.501644	-1.336135	1.301642	0.000907999	0.03954841
gene:Solyc12g014450.1	279.6693	-1.332377	0.293451	5.90E-07	0.000159669
gene:Solyc01g105890.2	54.38722	-1.31666	0.8691858	0.000650789	0.03152857
gene:Solyc07g048060.1	60.2612	-1.314048	0.91803983	0.000723432	0.0334619
gene:Solyc08g075790.2	205.9035	-1.309971	0.63967653	0.000287824	0.01819334
gene:Solyc10g086250.1	106.8644	-1.301925	1.30302763	0.000979258	0.04102323
gene:Solyc08g074630.1	259.6487	-1.297745	1.0922348	0.000930424	0.03992278
gene:Solyc12g049350.1	21.08583	-1.294812	1.59076208	0.000891832	0.03912522
gene:Solyc06g062960.1	237.9816	-1.294118	0.58473093	0.000217329	0.015426
gene:Solyc09g072710.2	308.4399	-1.290344	0.3659893	1.33E-05	0.002025766
gene:Solyc02g087800.2	55.67929	-1.289602	0.47307222	8.12E-05	0.007699371
gene:Solyc11g020820.1	148.3351	-1.288386	0.3344741	5.46E-06	0.001005243
gene:Solyc03g123540.2	14.8948	-1.288348	1.03791155	0.000914332	0.03962415
gene:Solyc09g007260.2	462.3665	-1.28217	0.86791828	0.000723585	0.0334619
gene:Solyc07g061800.2	423.815	-1.27886	1.54703606	0.000943433	0.04007245
gene:Solyc08g069140.2	342.053	-1.273874	0.39557032	2.77E-05	0.00361343
gene:Solyc05g024160.2	209.5671	-1.26104	0.41048121	3.86E-05	0.004519288
gene:Solyc09g015000.2	33.35463	-1.254792	0.56429135	0.000218339	0.01544637
gene:Solyc03g007240.2	1336.116	-1.240466	0.4650315	9.16E-05	0.008255814
gene:Solyc01g091360.2	416.2305	-1.240458	0.56123715	0.000225614	0.01570112
gene:Solyc12g006460.1	1748.641	-1.233822	0.80342738	0.000722635	0.0334619
gene:Solyc01g009570.2	161.1153	-1.22713	0.77503494	0.00068013	0.03243501
gene:Solyc10g052490.1	320.5179	-1.226047	0.82654497	0.000787604	0.03565076
gene:Solyc05g041420.2	193.8574	-1.224483	0.20575771	6.11E-10	4.84E-07
gene:Solyc01g098240.1	11.57727	-1.22358	0.9806521	0.001038061	0.04265034
gene:Solyc04g081290.2	846.1981	-1.216566	0.65136181	0.000429766	0.02348324
gene:Solyc10g008840.2	113.4682	-1.209975	0.59507888	0.000319827	0.01924815

gene:Solyc03g007340.2	11.83654	-1.19983	1.42756952	0.001164485	0.04615809
gene:Solyc08g066450.1	52.7802	-1.188142	0.5342064	0.000224455	0.0156715
gene:Solyc00g009120.2	50.54036	-1.183265	0.43650762	8.25E-05	0.007699371
gene:Solyc12g076320.1	36.93039	-1.181	0.40899266	5.58E-05	0.005964039
gene:Solyc01g058260.2	1802.874	-1.179699	0.37018295	2.77E-05	0.00361343
gene:Solyc06g082030.2	26.09326	-1.175469	0.81066897	0.00089456	0.0391645
gene:Solyc07g042540.2	10.52374	-1.169047	1.20581097	0.001330963	0.04988775
gene:Solyc10g050450.1	11.16551	-1.163342	0.89741938	0.001113468	0.04473135
gene:Solyc08g013820.2	57.15493	-1.153083	0.46369227	0.000131863	0.01104807
gene:Solyc03g098760.1	19.8772	-1.152156	1.85199441	0.00068468	0.03243501
gene:Solyc03g122270.2	1307.41	-1.144505	0.18725758	1.98E-10	1.84E-07
gene:Solyc07g054630.2	48.3777	-1.141225	0.57100758	0.000355382	0.02064533
gene:Solyc03g118540.2	49.10217	-1.135135	0.58686744	0.000403968	0.02276712
gene:Solyc03g121100.2	13.76043	-1.134242	0.9055306	0.001235717	0.04835366
gene:Solyc02g064620.2	305.8175	-1.127755	0.43141339	9.94E-05	0.008808819
gene:Solyc02g091030.2	404.723	-1.126931	0.35899937	2.92E-05	0.003745812
gene:Solyc06g005910.2	141.1927	-1.120013	0.66350171	0.000649319	0.03152857
gene:Solyc06g076670.2	710.1365	-1.115025	0.64822387	0.00061502	0.03048699
gene:Solyc06g066650.2	218.9014	-1.112457	0.39247488	5.97E-05	0.006343678
gene:Solyc01g080640.2	4672.287	-1.104818	0.49152336	0.000216607	0.015426
gene:Solyc06g068700.2	21.53383	-1.099495	0.62482956	0.000578381	0.02937373
gene:Solyc01g098320.2	104.8029	-1.091427	0.39203526	6.58E-05	0.006694976
gene:Solyc05g010540.2	126.0298	-1.087954	0.2769539	2.83E-06	0.000622367
gene:Solyc03g097170.2	105.0364	-1.084961	0.68752644	0.000820991	0.0369273
gene:Solyc03g083620.1	632.2912	-1.077305	0.79111526	0.001188146	0.0469219
gene:Solyc07g063910.2	404.4276	-1.07573	0.80796968	0.001245199	0.04854686
gene:Solyc02g091140.2	849.4633	-1.071772	0.34787104	3.10E-05	0.003853203
gene:Solyc02g085870.2	4184.265	-1.07062	0.32248372	1.65E-05	0.002377221
gene:Solyc12g099000.1	2923.397	-1.064723	0.64450878	0.000730489	0.03363555
gene:Solyc02g088770.2	54.51068	-1.060455	0.5004741	0.000284224	0.01812672
gene:Solyc00g006820.2	1078.232	-1.059627	0.70290344	0.000958153	0.04053072
gene:Solyc09g031790.2	120.1469	-1.058097	0.48341027	0.000244783	0.01660248
gene:Solyc01g091850.2	33.7035	-1.0569	0.79030536	0.001272961	0.04873982
gene:Solyc02g070530.2	256.275	-1.037628	0.4540419	0.00019742	0.01465398
gene:Solyc11g066680.1	316.7362	-1.023456	0.73833495	0.001238136	0.04835975
gene:Solyc04g074270.2	2170.921	-1.02243	0.50930862	0.000361436	0.02092139
gene:Solyc01g080700.2	223.9018	-1.020267	0.4742587	0.000264354	0.0173782
gene:Solyc02g068030.1	755.733	-1.019998	0.7490701	0.001296002	0.04931359
gene:Solyc12g036120.1	80.67339	-1.004936	0.3641058	6.30E-05	0.006467985
gene:Solyc12g027800.1	84.26658	-1.004643	0.33313779	3.26E-05	0.003956295

Sup Table 6: Primers

Primer name	Sequence (5'-3')	Purpose
MSssI_PCR_For	AAGTTGAGAACAAGACTAAGAA	Detection of methylase gene in transgenic plants
MSssI_PCR_Rev	GTTCTTGAGAGTTCTCTTATAA	
MSssIN-IV-R	AGGTAGAAGCAGAAACTTACCTGCGAATGCTTCGAACAC	Cloning of the potato *IV2* intron
MSssIN-IV-F	GTGTTCGAAGCATTCGCAGGTAAGTTTCTGCTTCTACCT	
MSssIC-IV-R	CTTTTCTTTGTGCACCTATACCTGCACATCAACAAATTTTG	
MSssIC-IV-F	CAAAATTTGTTGATGTGCAGGTATAGGTGCACAAAGAAAAG	
MSssI_HindIII For	CGCAAGCTTATGTCTAAAGTTGAGAACAAG	Cloning of methylase
MSssI_HindIII Rev	CGCAAGCTTTCACACCTTCCTCTTCTTC	
Lhg4_453_FWD	TGACCAGACACCCATCAACAGTA	Detection of *Lhg4* transgene
Lhg4_1004_rev	GGCGAATTCCGATCCACTAG	
qRT-TIP41-Fwd	ATGGAGTTTTTGAGTCTTCTGC	qRT-PCR
qRT-TIP41-Rev	GCTGCGTTTCTGGCTTAGG	
qRT-MSssI-Fwd	AGCATTCGCAGGTATAGGTGC	qRT-PCR
qRT-MSssI-Rev	CTTCCAATATCCGTTAGACACAG	
